# The Emergence of Resistance to Fungicides

**DOI:** 10.1371/journal.pone.0091910

**Published:** 2014-03-21

**Authors:** Peter H. F. Hobbelen, Neil D. Paveley, Frank van den Bosch

**Affiliations:** 1 Rothamsted Research, Harpenden, Hertfordshire, United Kingdom; 2 ADAS UK Ltd, High Mowthorpe, Duggleby, Malton, North Yorkshire, United Kingdom; Louisiana State University, United States of America

## Abstract

Many studies exist about the selection phase of fungicide resistance evolution, where a resistant strain is present in a pathogen population and is differentially selected for by the application of fungicides. The emergence phase of the evolution of fungicide resistance - where the resistant strain is not present in the population and has to arise through mutation and subsequently invade the population - has not been studied to date. Here, we derive a model which describes the emergence of resistance in pathogen populations of crops. There are several important examples where a single mutation, affecting binding of a fungicide with the target protein, shifts the sensitivity phenotype of the resistant strain to such an extent that it cannot be controlled effectively (‘qualitative’ or ‘single-step’ resistance). The model was parameterized for this scenario for *Mycosphaerella graminicola* on winter wheat and used to evaluate the effect of fungicide dose rate on the time to emergence of resistance for a range of mutation probabilities, fitness costs of resistance and sensitivity levels of the resistant strain. We also evaluated the usefulness of mixing two fungicides of differing modes of action for delaying the emergence of resistance. The results suggest that it is unlikely that a resistant strain will already have emerged when a fungicide with a new mode of action is introduced. Hence, ‘anti-emergence’ strategies should be identified and implemented. For all simulated scenarios, the median emergence time of a resistant strain was affected little by changing the dose rate applied, within the range of doses typically used on commercial crops. Mixing a single-site acting fungicide with a multi-site acting fungicide delayed the emergence of resistance to the single-site component. Combining the findings with previous work on the selection phase will enable us to develop more efficient anti-resistance strategies.

## Introduction

The evolution of fungicide resistance can be divided into an emergence phase and a selection phase [1], [2], [3]. In the emergence phase, the resistant strain has to arise through mutation and subsequently invade the pathogen population. In this phase, the number of fungicide resistant lesions is very small and the resistant strain may become extinct due to stochastic variation, in spite of fungicide applications providing the resistant strain with a higher fitness than the sensitive strain. The length of the emergence phase (emergence time) can be defined as the time from the introduction of a new fungicide mode of action until the resistant strain succeeds in building up a large enough sub-population so that it is unlikely to die out due to chance. The evolution of resistance then enters the selection phase in which the application of fungicides increases the frequency of the resistant strain in the pathogen population [1], [3].

**Figure 1:**
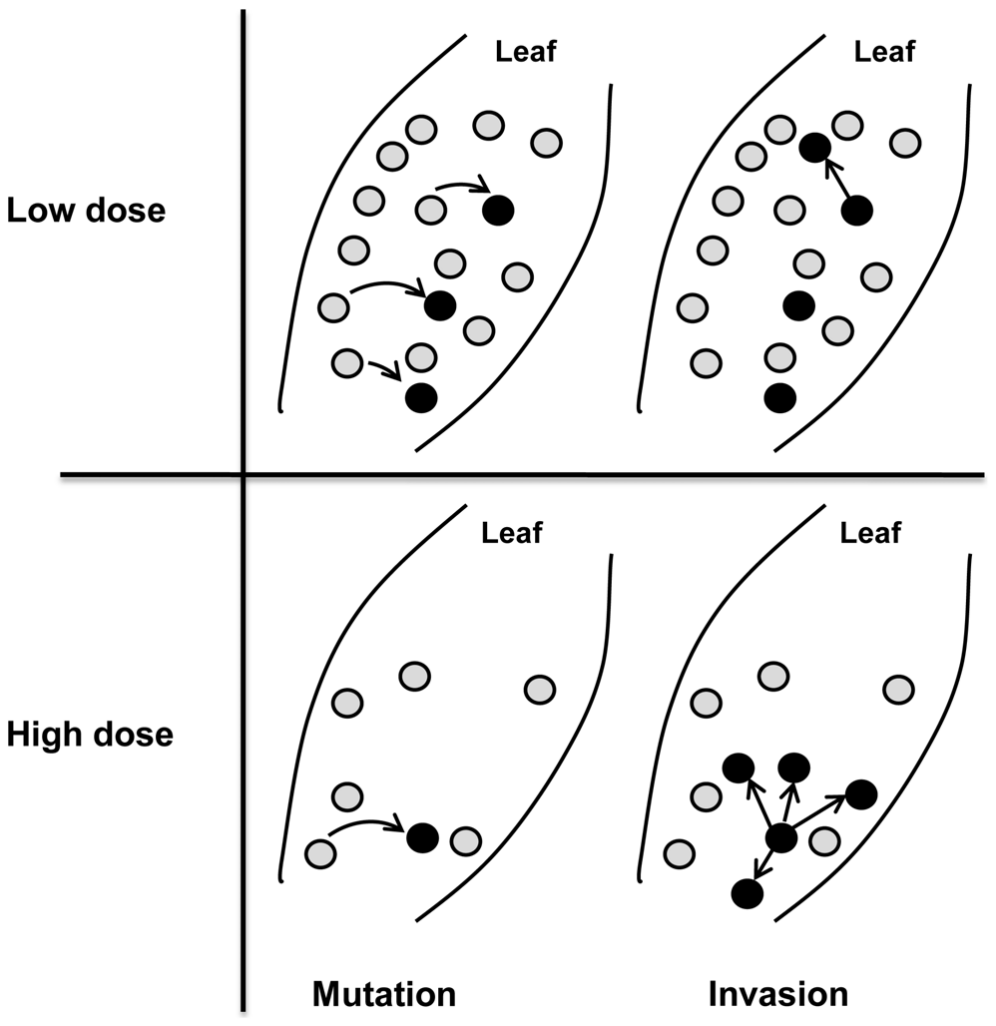
Fungicide dose and resistance emergence. The effect of the dose rate of a fungicide on the rate at which resistant lesions (black circles) arise through mutation and subsequently invade a sensitive pathogen population (grey circles). Curved arrows in the left subfigures represent mutation events and straight arrows in the right subfigures represent the colonization of new leaf area by the resistant lesions that arose through mutation in the left subfigures. This figure was adapted from figure 8 in [3].

**Figure 2:**
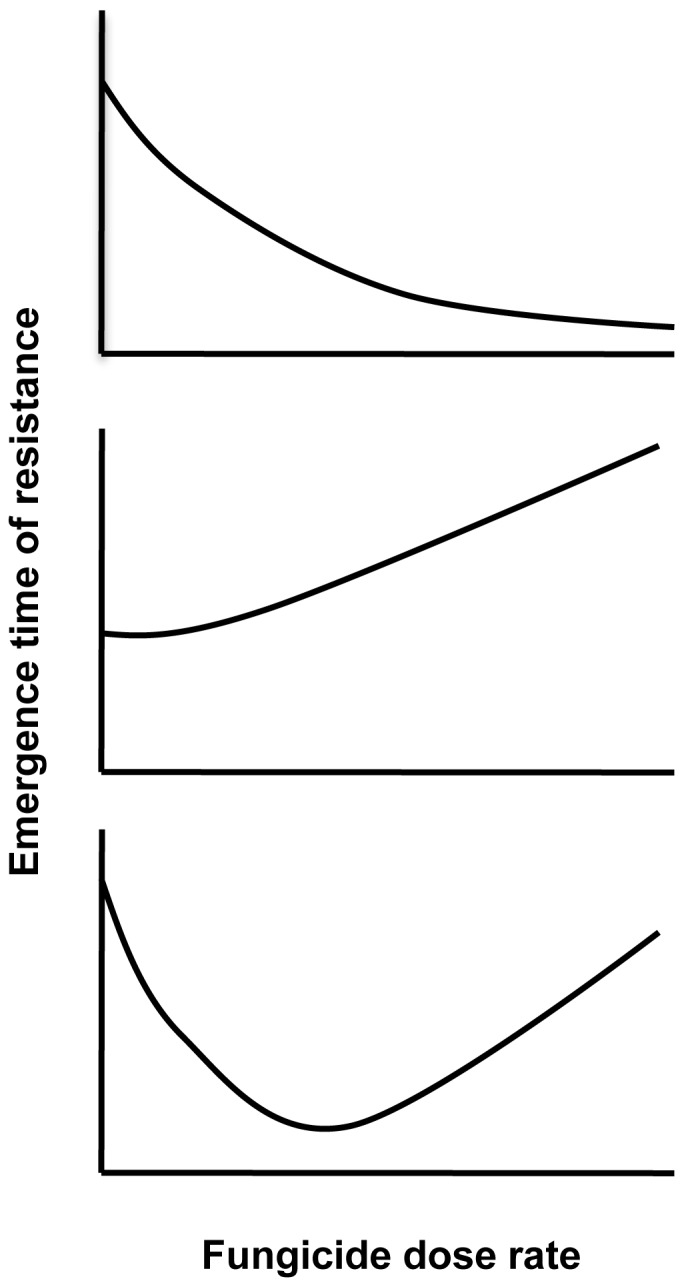
The shape of dose-emergence time curves. Possible ways in which the dose rate of a high-risk fungicide may affect the emergence time of resistance in a sensitive pathogen population. This figure was adapted from figure 9 in [3].

Fungicide resistance management strategies aim to delay the evolution and spread of resistance in a sensitive pathogen population, while ensuring effective disease control. Due to the differences in the dynamics of the resistant strain between the emergence phase and the selection phase, the usefulness of resistance management strategies may also differ between these two phases. For example, in the selection phase, the frequency of resistance in the pathogen population will generally increase faster for higher dose rates of the fungicide [3]. However, in the emergence phase, there are two opposing effects of dose on resistance evolution: A high dose rate of a fungicide (close to, or at the label recommended dose) may delay the emergence of resistance by reducing the size of the sensitive pathogen population and therefore the number of resistant mutants produced per unit time. However, the smaller pathogen population will reduce the competition between the sensitive and the resistant strain for healthy host tissue to infect and may therefore increase the probability that the resistant mutant invades the pathogen population (Fig. 1). We therefore hypothesize that the choice of dose rate of a fungicide in the emergence phase may change the emergence time in a number of different ways (Fig. 2). If the emergence time is most sensitive to changes in the number of mutations produced per time unit, the emergence time will increase with increasing dose rate of the fungicide. However, if the emergence time is most sensitive to changes in the strength of competition for healthy leaf area, the emergence time will decrease with increasing dose rate of the fungicide.

There is a range of experimental studies on the development of resistance in response to the dose rates of a fungicide and the mixing or alternation of fungicides [4], [5], [6], [7], [8], [9], [10], [11], [12]. However, in many of these studies resistant strains were either introduced [4], [6], [13] or were already present at a significant frequency at the start of experiments [7], [9], [14]. As even a frequency of 1% represents a large population of resistant lesions, these studies describe the selection phase in the evolution of fungicide resistance. The effect of fungicide treatment strategies on emergence time can therefore not be determined from the experimental literature.

There are some papers in the biomathematical literature studying the emergence of, what is called, escape mutants [15], [16], [17]. The models and methods developed give insight into the life-cycle parameters that are of key relevance for the emergence of new pathogen strains. It is however not possible to use these results to study the emergence of fungicide resistance due to the absence of seasonality in host density, and the fact the selection pressure (the fungicide) is not constant through time. Previously we have shown that in the selection phase this periodicity of the host density and the time dependence of the selection pressure are key to understand both the qualitative and quantitative relation between selection for fungicide resistance and fungicide application regimes [1], [18], [19], [20].

To our knowledge, no models have been published that account for the time dependence of key processes as well as the stochastic nature of resistant mutants arising and reproducing to invade a sensitive pathogen population in the emergence phase of the evolution of fungicide resistance. This also holds for models of insecticide and herbicide resistance. The aim of this study was therefore to develop a model for the emergence phase in the evolution of fungicide resistance, which describes the effect of fungicides on mutation and invasion. The model was derived from a successfully tested fungicide resistance model [20] describing the section phase and then parameterized for *Mycosphaerella graminicola* on winter wheat (*Triticum aestivum*).

To show how this model could be used to evaluate resistance management strategies, we determined the effect of the dose rate of a high resistance risk fungicide on the emergence time of resistance in a population of *M. graminicola* on winter wheat for different mutation probabilities, fitness costs of resistance and sensitivity levels of the resistant strain. We also evaluated the usefulness of mixing a high-risk fungicide with a low-risk fungicide for delaying the emergence of resistance. For the analyses in this paper we define a high-risk fungicide as a fungicide prone to substantial efficacy reduction due to a single mutation in the pathogen strain, such that selection for the resistant strain will eventually result in ineffective disease control by the high-risk fungicide used alone. We define a low-risk fungicide as a fungicide for which resistance does not evolve in the pathogen population in the time frame under consideration, but with efficacy that is too low to provide sufficient disease control on its own. These high and low-risk fungicides might typically represent single-site and multi-site acting substances.

## Materials and Methods

### Type of resistance described by the model

We developed a model to describe the emergence of resistance to high-risk fungicides which are prone to substantial efficacy reduction due to a single mutation in the pathogen strain. We assume that this single mutation decreases the sensitivity of the resistant pathogen strain to such an extent that the high-risk fungicide loses its ability to provide sufficient disease control of a pathogen population dominated by the resistant strain. The average difference in sensitivity between the sensitive and resistant pathogen populations is then much larger than the difference in sensitivity within these two populations. In that case, it is reasonable to represent the pathogen population as consisting of one sensitive and one resistant strain. This type of qualitative resistance development has occurred in response to, for example, methyl benzimidazole carbamate (MBC) [21] and quinone outside inhibitor (QoI) fungicides [22], [23].

### An overview of the model structure

**Figure 3:**
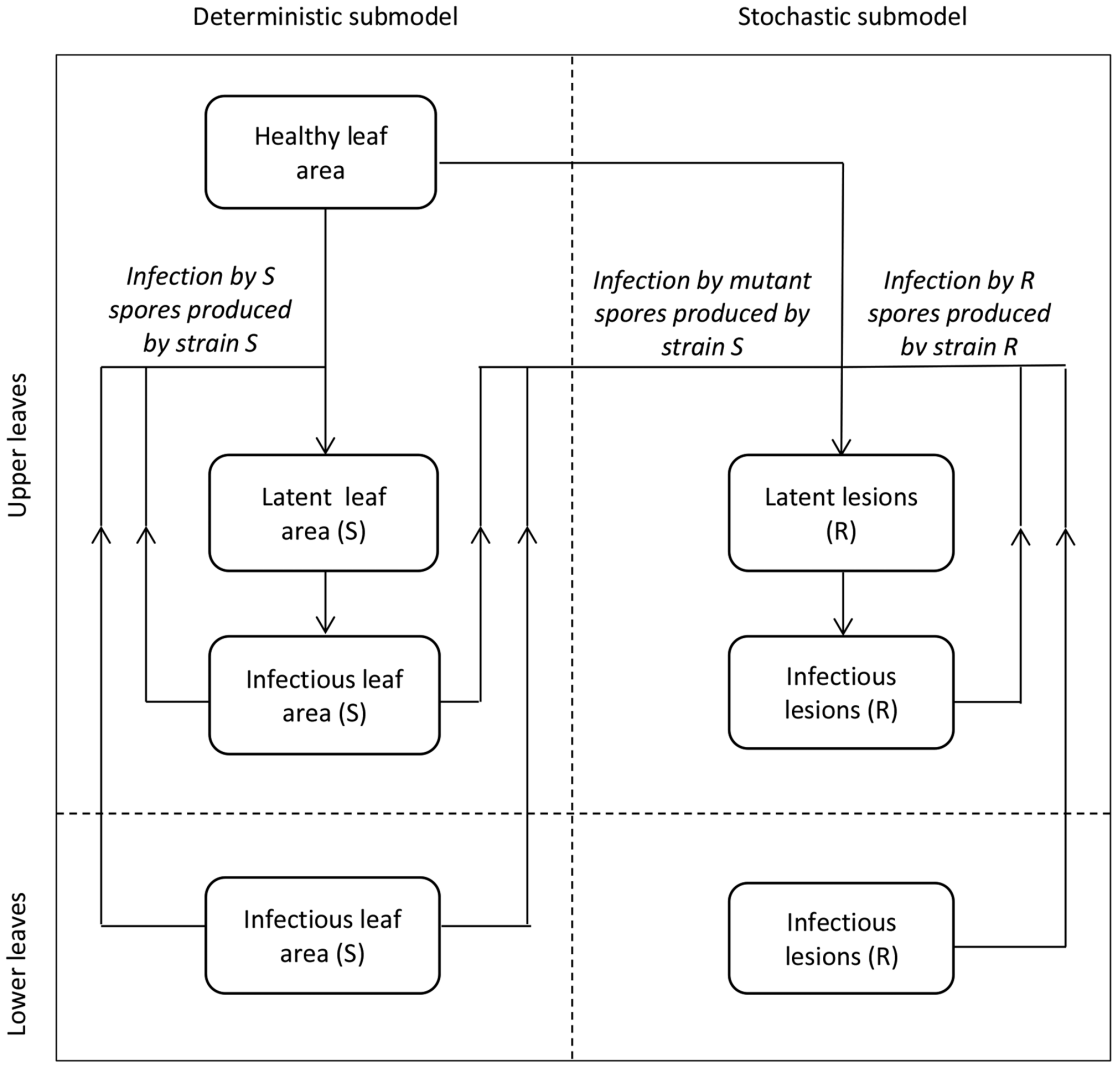
The structure of the simulation model. The model describes the emergence of a resistant pathogen strain (R) in a sensitive population (S) of *M. graminicola* on winter wheat in response to applications of a high-risk fungicide.

The model describes a resistant strain arising by mutation and reproduction in a sensitive population of *M. graminicola* on winter wheat in response to solo use of a high-risk fungicide or mixtures of a high-risk and a low-risk fungicide. The main part of the model, which describes the emergence of resistance within growing seasons, consists of a deterministic and a stochastic sub-model (Fig. 3). The deterministic sub-model describes the dynamics of the crop canopy, the sensitive pathogen strain and the variation in the fungicide concentrations in time. This part of the model was derived from a successfully tested fungicide resistance model for the selection phase of fungicide resistance development [20]. It was not necessary to use a stochastic sub-model to describe the seasonal dynamics of the sensitive strain, because the sensitive stain is always present in high enough densities to prevent extinction due to random processes and is well represented by the mean of the process. Using a deterministic sub-model had the advantage of a much shorter simulation times. A stochastic sub-model was used to describe the dynamics of the resistant strain, because the population of resistant lesions is very small and random processes may lead to the extinction of this strain. We assume that the frequency of the resistant strain in the pathogen population is too small to affect the dynamics of the sensitive strain through competition for healthy leaf area.

### The structure of the deterministic sub-model

See Figure 3 for a graphical presentation of the model structure.

#### The dynamics of the crop canopy

The model predicts the seasonal dynamics of the canopy in order to account for the availability of healthy leaf area on the growth of the pathogen population. The canopy consists of the combined areas of leaves 1–3 (counting down from the flag leaf, which is designated leaf 1), because this leaf area intercepts the sprayed fungicides. Hereafter, we refer to leaves 1–3 as the “upper leaves” and refer to leaves further down the stem as “lower leaves”. We use the term “density” to refer to leaf area per area of ground. The density of the total leaf area (*A*) is the sum of the densities of healthy, infected and dead leaf area and increases according to the monomolecular equation [24]:
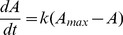
(1)


In the absence of disease, the seasonal dynamics of the healthy leaf area (*H*) consist of a growth phase, followed by a plateau and subsequently a senescence phase. The growth phase ends when the flag leaf has completely emerged (GS 39 on Zadoks' scale). Senescence starts at anthesis (GS 61) and is complete at the end of grain filling (GS 87). The density of healthy leaf area in the absence of disease is described by the equation
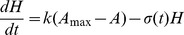
(2)where 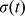 represents the senescence rate. The senescence rate increases exponentially from approximately 0 (<10^−7^) at GS 61 to a maximum value of 0.105 at GS 87 according to the function:
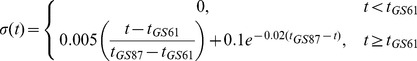
(3)


This reduces the healthy leaf area at GS 87 to < 1% of the maximum leaf area, which approximates complete senescence.

#### The sensitive pathogen population

The lifecycle of the sensitive pathogen strain is divided into a latent stage (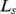) with length 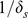 and an infectious stage (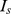) with length 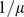. During the latent stage, the pathogen grows within the intercellular space in leaf tissues and senescence decreases the density of latent leaf area. At the start of the infectious stage, the pathogen kills the host cells and starts spore production. The rate at which infectious leaf area generates latent leaf area is determined by the product of i) the spore production rate per unit of infectious leaf area (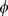), ii) the probability of a spore landing on the upper leaves of the canopy, iii) the probability of landing on healthy leaf area, given that a spore lands on the upper leaves 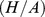, iv) the infection efficiency, and v) the area occupied by a lesion, which develops after the successful infection of healthy leaf area by one spore (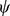). Points ii and iv are incorporated in compound parameter 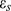. Hereafter, we refer to this parameter as the infection efficiency.

At the beginning of a growing season, the canopy becomes infected by spores from infectious lesions on lower leaves. The density of leaf area occupied by the infectious stage of the sensitive strain () decreases according to the function
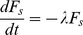
(4)with parameter 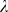 representing the loss rate of infectious leaf area on lower leaves. To constrain complexity, the model is not spatially explicit, hence spores produced by sensitive lesions have the same probability of landing on upper leaves (included in compound parameter ) whether they are produced on lower or upper leaves. This leads to the following equations to describe the dynamics of the sensitive pathogen population:
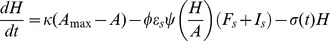
(5)

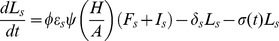
(6)

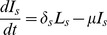
(7)


#### The impact of fungicides on the sensitive pathogen strain

Both the low-risk and high-risk fungicides were represented as having protectant activity, which reduced the infection efficiency of the sensitive strain (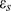). The high-risk fungicide was also represented as having eradicant activity. Eradicant activity was defined here as the ability of the fungicide to slow fungal growth during the latent period (fungistatic activity), rather than converting latent leaf area back into healthy tissue. Thus, symptom expression was delayed or prevented during the life of the crop canopy. This representation fits well with the observation that fungicides with ‘eradicant’ activity can only provide effective control of visible symptoms if treatments are applied prior to half way through the latent period. We therefore assumed that the eradicant activity of the high-risk fungicide increased the length of the latent stage of the sensitive strain (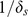). The infection efficiency depends on the concentrations of the low-risk (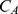) and high-risk fungicides (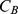) according to the function:
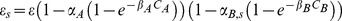
(8)


The length of the latent stage depends on the concentration of the high-risk fungicide according to the function:
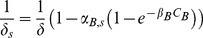
(9)


In these equations, parameters 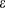 and 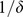 represent the infection efficiency and the length of the latent stage of the sensitive pathogen strain in the absence of fungicides, respectively. Parameter 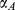 represents the maximum possible reduction of the infection efficiency by the low-risk fungicide and parameter 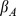 determines the curvature of the dose-response curve. Parameter 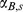 represents the maximum possible reduction of the life-cycle parameters of the sensitive strain by the high-risk fungicide and parameter 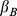 determines the curvature of the dose-response curve. The concentrations of the low-risk (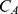) and high-risk fungicides (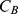) decay in time according to the functions:
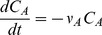
(10)

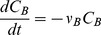
(11)with 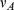 and 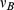 representing the decay rates of the low-risk and high-risk fungicides, respectively.

This leads to an exponential decay of the activity of the fungicides in time, with the loss of activity highest just after application of the fungicides. To explore the effect of the type of function used to describe the decay of the activity of fungicides on the emergence time, we also determined emergence times using a gamma distribution for the rate of fungicide decay in time. (Text S1). This change did not affect the qualitative conclusions about the effect of the dose rate of a high-risk fungicide on the emergence of resistance or the usefulness of mixtures of a low-risk and a high-risk fungicide for delaying the emergence of resistance to the high-risk fungicide (Text S1).

### Fungicide dose response curves

**Figure 4:**
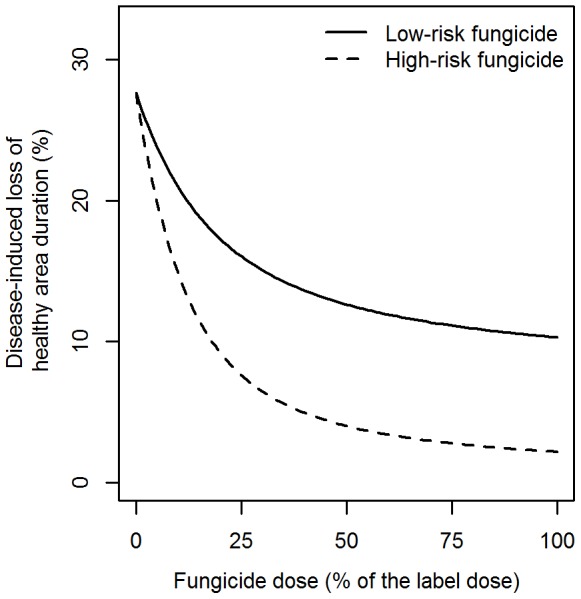
Fungicide dose response curves. The effect of the dose rate of the low-risk and high-risk fungicide (solo use) on the disease-induced loss of healthy area duration for a sensitive population of *M. graminicola* on winter wheat. Healthy area duration was calculated as the area under the green leaf area curve from anthesis to the end of the growing season and is an indicator of the yield loss of winter wheat [25]. We assumed an average epidemic of *M. graminicola* for the United Kingdom in the absence of fungicide applications. Fungicides were applied twice during a growing season (see text) at a constant dose rate.

Figure 4 shows the model predictions for the loss of healthy area duration, an indicator of yield [25], due to an average epidemic *M. graminicola* on winter wheat in the United Kingdom as a function of the dose rate in case of solo use of the low-risk fungicide and solo use of the high-risk fungicide for the scenario assuming exponential decay of fungicides.

### The structure of the stochastic sub-model

We used a modified version of Gillespie's stochastic simulation algorithm [26] to simulate the dynamics of the number of lesions of the resistant strain within a growing season. To use this algorithm, firstly, all possible events in the model which change the number of resistant lesions are labelled. Secondly, equations must be derived for the average rates at which these events occur at a given time *t* within a growing season. If *N* represents the total number of events, we labelled each event and corresponding event rate as *E*_i_ and *R*_i_ , respectively, with subscript i∈[1,*N*]. The modified version of Gillespie's stochastic simulation algorithm calculates the size of the next time step (*t*_step_) as the maximum step that can be taken while satisfying the following condition for the change in event rates (*R_i_*) during a time step:
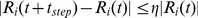
(12)


This condition limits the absolute change in any event rate 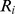 during a time step to a certain fraction 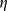 of the value of this event rate at time 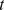 in order to ensure the accuracy of the stochastic simulation algorithm [26]. Parameter 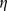 is conventionally set to 0. 03 [26] and we used this value in our simulations. The number of times that an event 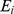 occurs during a time step is subsequently determined by drawing from a Poisson distribution with mean 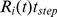. Below, we describe the events which change the number of lesions of the resistant strain and derive equations for the average rates of these events.

#### Events and event rates

The stochastic sub-model describes the variation in the number of lesions of the resistant pathogen strain in time. The life-cycle of the resistant strain is divided into a latent (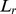) stage with length (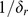) and an infectious stage (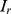) with length (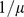). Latent lesions may die as a result of senescence. New latent lesions form as a result of infection by mutant spores from the sensitive strain and by spores from infectious lesions of the resistant strain on upper (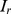) and lower leaves (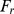). This leads to a total of six events, which can change the number of lesions of the resistant strain on upper and lower leaves.

The first event (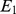) is the successful infection of healthy leaf area by a mutant spore produced by the sensitive strain. Mutant spores can be produced by the sensitive pathogen population on upper and lower leaves. The number of new latent lesions generated per time unit is determined by the product of i) the leaf area occupied by infectious lesions of the sensitive strain, ii) the spore production rate per unit of infectious leaf area (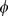), iii) the probability of a spore having the resistant genotype (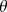) iv) the probability of a spore landing on the upper leaves of the canopy, v) the probability of landing on healthy leaf area, given that a spore lands on the upper leaves 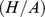, and vi) the infection efficiency of a resistant spore. The amount of infectious leaf area is determined by the product of the density of infectious leaf area (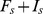) and the size of the wheat growing area (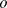). Points iv and vi are incorporated in compound parameter 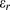. We assumed that spores produced by resistant lesions have the same probability of landing on upper leaves (included in compound parameter ) whether they are produced on lower or upper leaves. The average number of new latent lesions generated per time unit by infectious leaf area on both upper and lower leaves is then:
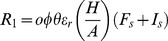
(13)


If this event occurs, the number of latent lesions of the resistant strain increases by one.

The second event (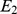) is the successful infection of healthy leaf area by a spore from an infectious lesion of the resistant strain. The average number of latent lesions generated per time unit by infectious lesions of the resistant strain on both upper and lower leaves is determined by the product of i) the total number of infectious lesions of the resistant strain (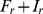), ii) the spore production rate per infectious lesion (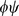), iii) the probability of a spore landing on the upper leaves of the canopy, iv) the probability of landing on healthy leaf area, given that a spore lands on the upper leaves 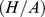 and v) the infection efficiency of a resistant spore. Points iii and v are incorporated in compound parameter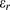. The average number of new latent lesions generated per time unit by infectious lesions of the resistant strain is then:
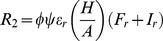
(14)


If this event occurs, the number of latent lesions of the resistant strain increases by one.

The third event (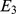) is the senescence of a latent lesion (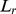). The average number of latent lesions which die per time unit as a result of senescence of green leaf area is
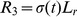
(15)with senescence rate 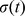. If this event occurs, the number of latent lesions of the resistant strain decreases by one.

The fourth event (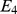) is the transition from a latent (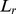) to an infectious lesion (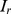). The average number of latent lesions which becomes infectious per unit of time is
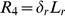
(16)with development rate 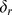 equal to the inverse of the length of the latent period. If this event occurs, the number of latent lesions of the resistant strain decreases by one and the number of infectious lesions of the resistant strain on upper leaves increases by one.

The fifth event (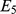) is the death of an infectious lesion on lower leaves (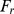). The average number of infectious lesions on lower leaves dying per unit of time as a result of reaching the end of the infectious period is
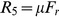
(17)with mortality rate 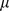 equal to the inverse of the length of the infectious period.

If this event occurs, the number of infectious lesions of the resistant strain on lower leaves decreases by one.

The sixth and last possible event (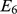) is the death of an infectious lesion on upper leaves (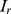). The average number of infectious lesions on upper leaves dying per time unit as a result of reaching the end of the infectious period is
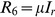
(18)with mortality rate 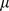 equal to the inverse of the length of the infectious period. If this event occurs, the number of infectious lesions of the resistant strain on upper leaves decreases by one.

#### Fitness costs of resistance and the impact of fungicides on the resistant strain

In the stochastic sub-model, which describes the dynamics of the resistant strain, fitness costs of resistance to the high-risk fungicide were assumed to reduce the infection efficiency of the resistant strain by a fraction 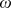. The protectant low-risk fungicide was assumed to reduce the infection efficiency of the resistant strain (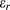). When resistance to the high-risk fungicide was represented as being partial (incomplete), the protectant activity of the high-risk fungicide reduced the infection efficiency and the eradicant activity of the high-risk fungicide increased the length of the latent stage of the resistant strain, 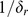. The infection efficiency of the resistant strain depends on the fitness costs of resistance and the concentrations of the low-risk (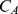) and high-risk fungicides (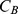) according to the function:
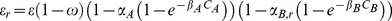
(19)


The length of the latent stage of the resistant strain depends on the concentration of the high-risk fungicide according to the function:
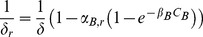
(20)


In these equations, parameter 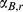 represents the maximum possible reduction of the life-cycle parameters of the partially resistant strain by the high-risk fungicide. In case of complete resistance, 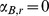.

#### The number of infectious lesions of the resistant strain at the start of a new growing season

The epidemic on the upper leaves is initiated by spores from infectious lesions on lower leaves. Which of these infectious lesions are resistant to the fungicide is determined by drawing from a binomial distribution 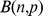 with mean 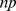 and variance 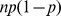. Parameter 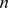 of the binomial distribution is the total number of infectious lesions on lower leaves at the start of a growing season:
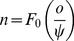
(21)


Parameter 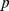 of the binomial distribution is the probability of an infectious lesion on lower leaves being resistant to the fungicide, at the start of a growing season. This probability was set to the fraction of infectious lesions at the end of the previous growing season, which was resistant to the high-risk fungicide:
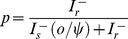
(22)


In the emergence phase the frequency of resistance (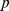) is very low and 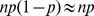. As a result the mean and variance of the binomial distribution are approximately the same and amount to:
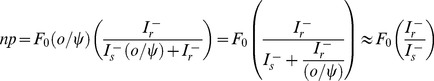
(23)


This simplification can be made, because the number of resistant lesions at the end of the previous growing season (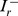) is much smaller than the ratio of the total wheat growing area and the area occupied by a single lesion, 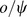. It follows that the size of the wheat growing area (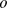) has a negligible effect on the number of infectious lesions of the resistant strain at the start of the next growing season, because it does not occur in the approximation on the right side of the equation.

### Parameter values

The definitions, values and dimensions of the model parameters are given in Table 1. The values of all parameters were the same as we have used before [18], [19], [20], except for the spore production rate per infectious leaf area (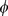), the area occupied by one *M. graminicola* lesion (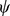), the infection efficiency (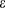), (previously multiplied to give the transmission rate, but for the stochastic sub-model included as separate parameters), the size of the wheat growing area (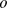) and the probability that a spore produced by the sensitive strain carries a resistant mutation (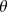).The spore production rate per infectious leaf area was calculated by dividing the total number of spores produced during the infectious period per infectious leaf area [27] by the length of the infectious period in degree- days [18]. The area occupied by one *M. graminicola* lesion (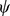) was taken from [28]. The product of the infection efficiency (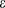), the spore production rate per infectious leaf area (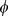) and the area occupied by one *M. graminicola* lesion (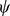) has the same value as the transmission rate parameter (ρ) in [18]. The infection efficiency (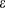) was therefore calculated as 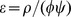. We set the size of the wheat growing area to 350,000 km^2^, which reflects the size of the winter wheat growing area in Europe during the years 2000–2008 (Eurostat, the statistical office of the European Union). We subsequently chose the value of parameter 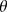 such that the median emergence time at half dose rates of the fungicide amounted to ten years for the default scenario (see below). This resulted in mutation probability 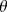 amounting to 1.13•10^−16^. We used ten years, because this is an average time for the period from the introduction of a high-risk fungicide on the market until the first detection of resistant strains in European crops [29]. It should be noted that the emergence of resistance is influenced by the product of the size of the parameters 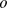 and 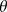 (Eq. 13). There are many combinations of the values of parameters 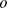 and 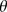 which result in the same product. To avoid confusion, it should be noted that the mutation probability 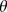 is not the same as the mutation rate. In this paper, we define the mutation rate as the number of mutant spores with a resistant genotype produced per time by the sensitive pathogen population.

**Table 1:** The definitions, values and dimensions of model parameters^a^.

Parameters	Definition	Value	Dimension
*_Host_*			
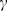	Growth rate of leaf area	1.26•10^−2^	t^−1b^
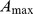	Maximum density of leaf area	4.1	leaf area per area of ground
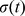	Senescence rate	Eq. 3	t^−1^
	The size of the wheat growing area	3.5•10^5^	km^2^
*_All pathogen strains_*			
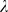	Loss rate of infectious leaf area/lesions on lower leaves^c^	8.5•10^−3^	t^−1^
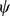	The area occupied by one lesion	0.3•10^−10^	km^2^
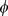	Spore production rate per unit of infectious leaf area	7.3•10^12^	t^−1^ km^−2^
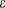	Infection efficiency in the absence of fungicides and fitness costs of resistance^d^	9.5•10^−5^	-e
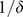	Length of the latent stage in the absence of fungicides	266	T
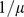	Length of the infectious stage	456	T
*_Sensitive pathogen strain_*			
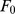	Initial density of infectious lesions on lower leaves^c^	1.09•10^−2^	leaf area per area of ground
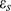	Infection efficiency in the presence of fungicides	Equation 8	t^−1^
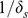	Length of the latent stage in the presence of fungicides	Equation 9	T
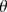	Mutation probability	Variable^f^	-e
*_Resistant pathogen strain_*			
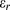	Infection efficiency in the presence of fungicides and/or fitness costs of resistance	Equation 18	t^−1^
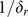	Length of the latent stage in the presence of fungicides	Equation 19	T
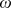	The fraction by which the infection efficiency of the resistant strain is reduced due to fitness costs of resistance	Variable^f^	-e
*_Dose-response curve and decay rate parameters_*			
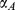	Maximum reduction of the infection efficiency of the sensitive and resistant strain by the low-risk fungicide	0.48	-e
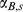 _, _ 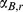	Maximum reduction of the life-cycle parameters of the sensitive ( 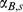 ) and resistant strain ( 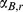 ) by the high-risk fungicide	1, variable^f^	-e
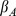 _, _ 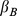	Curvature parameter of the dose-response curve for the low-risk ( 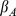 ) and high-risk fungicide ( 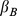 )	9.9, 9.6	-e
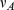 _, _ 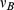	Decay rate of the low-risk ( 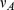 ) and high-risk fungicide ( 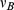 )	6.9•10^−3^, 1.1•10^−2^	t^−1^

a Parameter values were taken from [18], except for parameters , 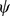, 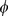 and 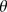. The estimation of the values of these parameters is described in the text.

b The character ‘t’ represents degree-days.

c Lower leaves are leaves that emerged before leaf 3, when counting down from the flag leaf (flag leaf  =  1).

d A compound parameter which combines the infection efficiency and the probability of a spore landing on the upper leaves of the canopy (see text).

e Dimensionless.

f See the text for the range of values of parameters 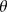, 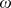 and 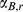 in the simulations.

### Emergence criterion

We define the emergence time as the number of growing seasons from the introduction of a new fungicide mode of action on the market until the resistant subpopulation has reached a size that makes extinction due to stochastic processes unlikely. We used a fixed emergence threshold of 30 resistant lesions at the start of a growing season to determine whether the size of the resistant subpopulation was large enough to have emerged. At or above this emergence threshold, the probability of the resistant strain becoming extinct during a period of 100 years in the absence of new mutations varied between 0–3%, depending on the simulated scenario (see below). To assess the accuracy of this emergence threshold we defined an alternative emergence threshold and compared results. For the alternative, we calculated emergence thresholds specific for each scenario as the lowest possible number of resistant lesions at the start of a growing season for which the probability of the resistant strain becoming extinct during a period of 100 years in the absence of new mutations was < 5%. Emergence times were very similar for both types of emergence thresholds (Text S1). The emergence times for all simulations in the main text are determined using a fixed threshold of 30 resistant lesions at the start of a growing season. This corresponds to a frequency of resistance in the total pathogen population at the start of the growing season of 2.4·10^−11^%.

### Simulations

Having parameterized the model, we first simulated the dynamics of the resistant strain during a 1000-year period before the introduction of the fungicide to study the possibility that a resistant strain might already be present in the pathogen population at numbers above the emergence threshold, prior to the introduction of the fungicide on the market. We subsequently determined the effect of the dose rate of the high-risk fungicide on the emergence time of the resistant strain, for different values for the mutation probability, fitness costs of resistance and the sensitivity of the resistant strain to the fungicide. In all scenarios, the high-risk fungicide was applied twice during each growing season which corresponds to a standard UK treatment programme [30]. The first spray each season was applied at the full emergence of leaf 3 (approximately GS 32) and the second spray was applied at complete emergence of leaf 1 (GS 39), counting down from the flag leaf. For each scenario, we varied the dose rate of the high-risk fungicide from 10% to 100% of the label recommended dose in steps of 10% and performed 5000 simulations per dose rate. The median emergence time was stable for this number of repetitions (Text S1).

We first determined the effect of the dose rate of the high-risk fungicide on the emergence time for the default scenario. In the default scenario, i) fitness costs were assumed to reduce the infection efficiency of the resistant strain by 10%, ii) resistance to the high-risk fungicide was assumed to be complete, and iii) the mutation probability was chosen such that the median emergence time at half dose rates was 10 years. To determine the effect of variations in the mutation probability on the emergence time, we performed simulations with a mutation probability amounting to 0.1, 0.2, 5 and 10 times the mutation probability in the default scenario. To determine the effect of variation in the fitness costs of resistance on the emergence time, we performed simulations with the reduction of the infection efficiency increasing from 2.5 to 15% in steps of 2.5%, while keeping the values of other parameters the same as the default scenario. Finally, to determine the effect of variation in the sensitivity of the resistant strain to the high-risk fungicide, we performed simulations with the maximum reduction of life-cycle parameters by the high-risk fungicide increasing from 5 to 20% in steps of 5% (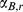 varying from 0.05 to 0.2 in steps of 0.05, respectively), while keeping the values of other parameters the same as the default scenario.

We also determined the effect of mixing a low-risk with a high-risk fungicide on the emergence time of resistance for the default scenario only. Both fungicides were applied twice during each growing season, as described above. We varied the dose rates of both the low-risk and high-risk fungicides in the mixture from 10% to 100% of the label recommended dose in steps of 10%. We determined the emergence time for all possible combinations of these dose rates and performed 5000 simulations per combination of dose rates.

## Results

### The dynamics of the resistant strain in the absence of fungicides

**Figure 5:**
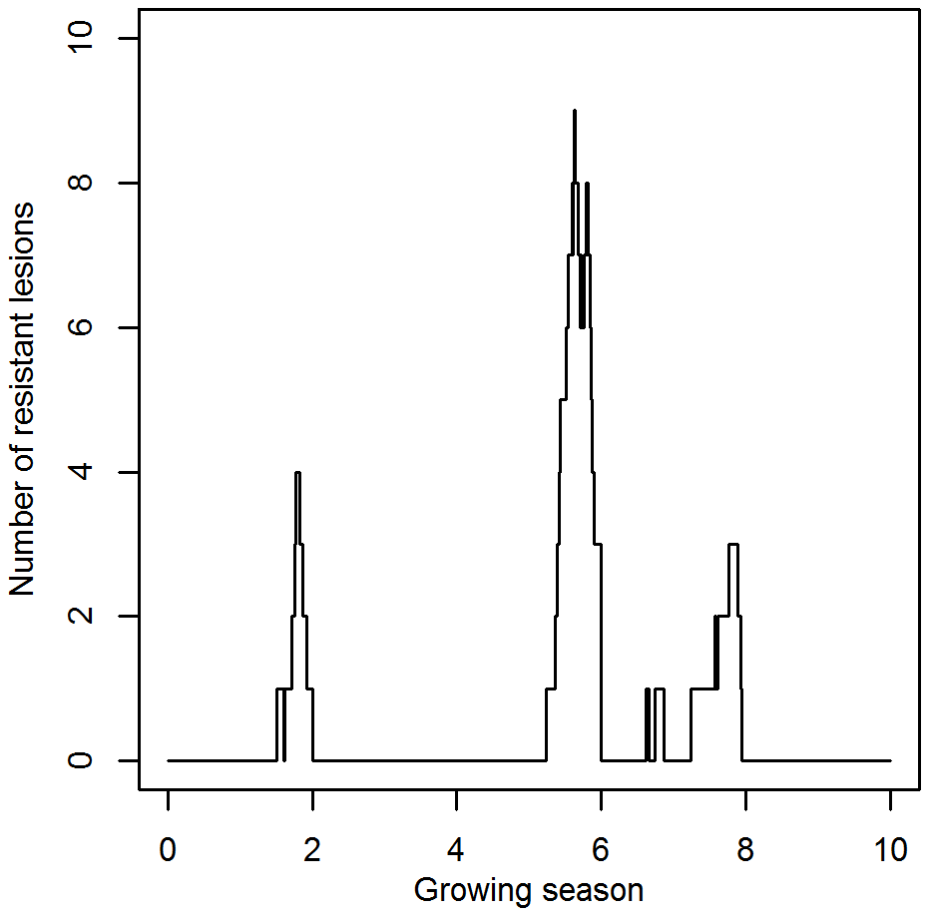
Temporal dynamics of the resistant sub-population. An example of the temporal dynamics of the number of resistant lesions in the absence of fungicide applications for the default scenario. In this scenario, the mutation probability amounts to 1.13•10^−16^ and fitness costs of resistance reduce the infection efficiency by 10%.

In the absence of fungicide, the resistant strain arises in the pathogen population through mutation, but fitness costs of resistance prevent it from building up a large number through drift. The temporal dynamics of the resistant strain in the absence of fungicides is characterized by the alternation of short periods during which the resistant strain is present, with periods during which the resistant strain is absent (Fig. 5). Increasing the mutation probability increases the percentage of time that the resistant strain is present in the pathogen population, while increasing the fitness costs of resistance decreases this percentage. The probability of the resistant strain having already emerged when a new fungicide mode of action is introduced equals the probability that the number of resistant lesions at the start of a growing season exceeds the emergence threshold (30 resistant lesions) in the absence of fungicides. This probability amounted to ≤ 0.16% for all simulated values of the mutation probability and fitness costs of resistance.

### The effect of the dose rate of the high-risk fungicide on the emergence time

**Figure 6:**
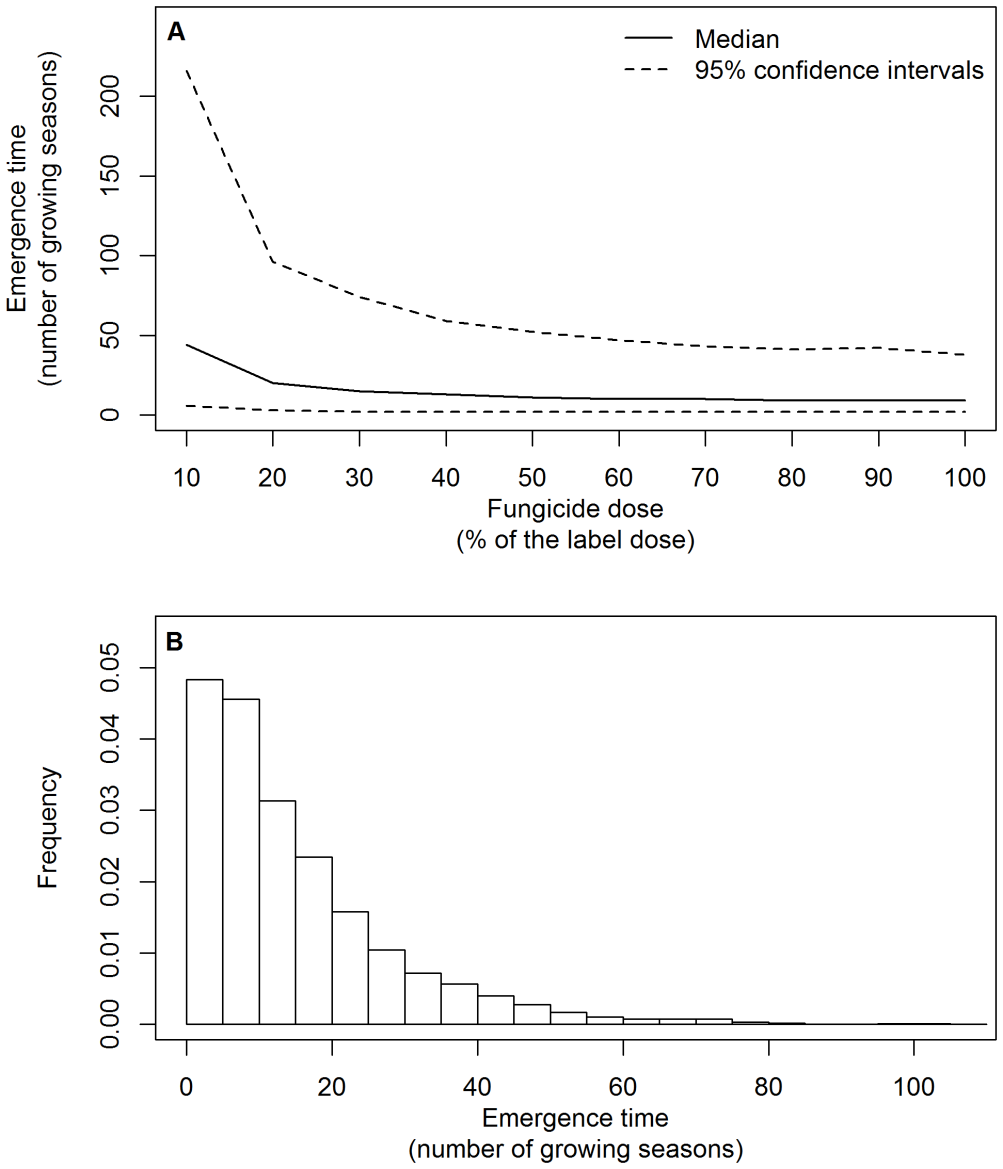
Emergence time and fungicide dose. The emergence time of a resistant strain in a sensitive population of *M. graminicola* on winter wheat in response to different dose rates of a high-risk fungicide for the default scenario (A). The bottom graph (B) shows the frequency distribution of the emergence time for a dose rate amounting to 50% of the label recommended dose. The shape of the distribution was the same for dose rates from 10 to 100% of the label recommended dose per spray. In the default scenario, the mutation probability was 1.13•10^−16^, fitness costs of resistance reduced the infection efficiency by 10% and resistance to the high-risk fungicide was complete.

We first determined the effect of dose rate of the high-risk fungicide on the emergence time for the default scenario, which assumes a 10% reduction of the transmission rate due to fitness costs of resistance, complete resistance to the fungicide and a mutation probability of 1.13•10^−16^. For this scenario, the median emergence time initially decreased sharply with increasing fungicide dose, but was much less sensitive to changes in dose when fungicide dose rates increased above approximately 50% of the label recommended dose (Fig. 6A). The distribution of the emergence time for a given dose rate was skewed to the right (Fig. 6B) and the size of 95% confidence interval of the emergence time was large with upper boundaries at least 38 years higher than lower boundaries.

### The sensitivity of the emergence time to changes in parameter values

**Figure 7:**
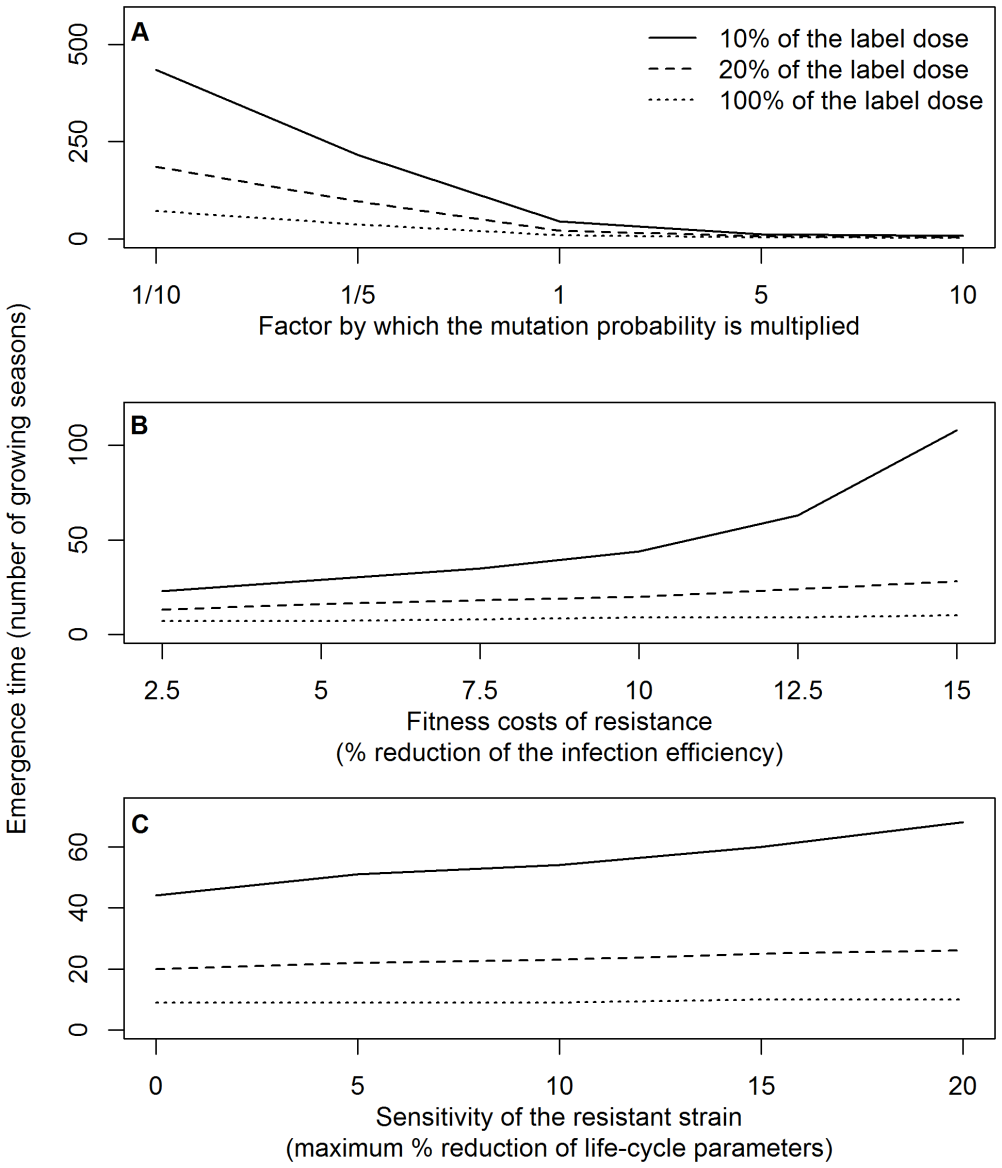
Emergence time and parameter values. The effect of the mutation probability (A), fitness costs of resistance (B) and the sensitivity of a resistant strain to a high-risk fungicide (C) on the emergence time of resistance in a sensitive population of *M. graminicola* on winter wheat. The emergence times are shown for dose rates of the high-risk fungicide amounting to 10, 20 and 100% of the label recommended dose per spray. The mutation probability is the probability that a spore produced by the sensitive pathogen population has a resistant genotype. By default, the mutation probability was 1.13•10^−16^, fitness costs of resistance reduced the infection efficiency by 10% and resistance to the high-risk fungicide was complete.

We subsequently determined the sensitivity of the emergence time to changes in the mutation probability, the fitness costs of resistance and the sensitivity of the resistant strain to the high-risk fungicide. Multiplying the mutation probability with a factor 10 decreased the median emergence time by a factor 4.3–6.3 while dividing the mutation probability by a factor 10 increased the median emergence time by a factor 7.9–9.9, depending on the dose rate of the high-risk fungicide (Fig. 7A). Changing the size of the wheat growing area has a similar effect on the emergence time as changing the mutation probability, because the emergence time is influenced by the product of both parameters. Decreasing the fitness costs of resistance from 10 to 2.5% (reduction of the infection efficiency of the resistant strain) decreased the emergence time by a factor 1.3–1.9, while increasing fitness costs of resistance from 10 to 15% increased the median emergence time by a factor 1.1–2.5, depending on the dose rate of the high-risk fungicide (Fig. 7B). The effect of varying the sensitivity of the resistant strain to the high-risk fungicide on the emergence time was determined by increasing the maximum possible reduction of the life-cycle parameters of the resistant strain by the high-risk fungicide from 0 (complete resistance) to 20%. This increased the median emergence time by a factor 1.1–1.6, depending on the dose rate of the high-risk fungicide (Fig. 7C). The emergence time was most sensitive to changes in these parameters for low dose rates of the high-risk fungicide. The size of the 95% confidence interval of the emergence time increased or decreased when the median emergence time increased or decreased due to the variation in parameter values described above.

For all parameter settings described above, varying the dose rate of the high-risk fungicide had the same qualitative effect on the median and 95% confidence interval of the emergence time as for the default scenario.

### The usefulness of mixing a low-risk with a high-risk fungicide to delay the emergence of resistance

Mixing a low-risk with a high-risk fungicide delayed the emergence of resistance to the high-risk fungicide in comparison to solo use of the high-risk fungicide at the same dose rate as in the mixture (Table 2). For a fixed dose rate of the high-risk fungicide, the delay in the emergence time initially increased with an increasing dose rate of the low-risk fungicide, but became much less sensitive when the dose rate of the low-risk fungicide increased above approximately 50% of the label recommended dose. For a fixed dose rate of the low-risk fungicide, the emergence time initially decreased sharply with an increasing dose rate of the high-risk fungicide, but was much less sensitive when dose rates of the high-risk fungicide increased above approximately 50% of the label recommended dose. The emergence time was delayed most by mixing the lowest dose rate of the high-risk fungicide with a dose rate of the low-risk fungicide higher than approximately 50% of the label recommended dose.

**Table 2:** The effect of mixing a low-risk and a high-risk fungicide^a^ on the number of growing seasons before resistance to the high-risk fungicide emerges^b^^,^ c in a population of *M. graminicola* on winter wheat.

Dose rate of the low-risk fungicide^d^	Dose rate of the high-risk fungicide^d^									
	10	20	30	40	50	60	70	80	90	100
0	46^e^	21^e^	15^e^	13^e^	11	10	9	9	9	8
10	55^e^	23^e^	17^e^	14	13	11	11	10	10	9
20	65^e^	25^e^	18	14	13	12	11	11	10	9
30	73^e^	27^e^	19	15	13	12	11	11	10	10
40	78^e^	27^e^	19	16	14	13	12	11	11	10
50	78^e^	29^e^	20	16	14	13	12	11	11	10
60	82^e^	29	20	16	14	13	12	11	11	11
70	80^e^	28	20	16	14	13	12	12	11	11
80	81^e^	28	20	16	15	13	12	11	11	11
90	81^e^	28	20	16	14	13	12	12	11	11
100	81^e^	28	19	16	14	14	12	12	11	11

a The low-risk fungicide was assumed to be not at-risk of resistance development, but unable to provide sufficient disease control when used alone. The resistant strain was assumed to be completely insensitive to the high-risk fungicide.

b The resistant strain was considered to have emerged when the number of resistant lesions reaches or exceeds a threshold (see text).

c The emergence times in the table were calculated for the default scenario, which assumes that i) fitness costs of resistance reduce the infection efficiency of the resistant strain by 10%, ii) resistance to the high-risk fungicide is complete and iii) a mutation probability amounting to 1.13•10^−16^.

d Fungicide doses are expressed as a fraction of the label recommended dose.

e Combinations of dose rates of the low-risk and high-risk fungicide that do not provide sufficient control of an average epidemic of *M. graminicola* on winter wheat. Effective disease control was defined as a disease-induced loss of healthy leaf area duration during the yield forming period equal to or below 5% [18].

## Discussion

To our knowledge, this is the first time that a model structure is presented to describe the emergence of resistance to high-risk fungicides in a pathogen population. The model consists of a deterministic sub-model to describe the dynamics of the host and the sensitive pathogen population. The resistant strain occurs in very low densities during the emergence phase and stochastic processes determine when resistant mutants arise and whether they survive or not. A stochastic sub-model was therefore used to describe the dynamics of the resistant strain. Although the model structure is generic and could be applied to many foliar patho-systems on determinate crops, we have as an example parameterized the model to describe the emergence of resistance in *M. graminicola* on winter wheat. For this specific system, we evaluated the effect of the dose rate of a high-risk fungicide on the emergence time of the resistant strain. We also determined the effect of mixing a high-risk fungicide with a low-risk fungicide on the emergence time of resistance to the high-risk fungicide. The model output suggests that the emergence time initially sharply decreases with increasing dose rate of the high-risk fungicide, but is virtually insensitive to changes within the range of higher dose rates typically needed for effective control of pathogens in commercial crops. This pattern was similar for a range of values for the mutation probability, fitness costs of resistance and sensitivities of the resistant strain to the high-risk fungicide. Mixing a high-risk fungicide with a low-risk fungicide delayed the emergence of resistance to the high-risk fungicide in comparison to solo use of the high-risk fungicide.

### Explanation of the model output

The initial sharp decrease in emergence time with increasing dose rate of the high-risk fungicide shows that the emergence time is more sensitive to a reduction in the competition for healthy leaf between the resistant and sensitive strain than to a reduction in the number of mutants generated per time unit. The sensitivity of the emergence time to changes in the dose rate of the high-risk fungicide is virtually negligible at higher dose rates. This effect of dose on emergence time is caused by the high curvature of the dose-response curve resulting in the asymptote being reached at quite a low fungicide dose. As a result, the impact of the high-risk fungicide on the density of the sensitive strain will be similar for dosages above 0.4. The number of mutants generated per time unit and the availability of healthy leaf area which both depend on the size of the sensitive pathogen population, are therefore also approximately constant for dose rates larger than 0.4. This explains the approximately constant emergence time for these dose rates.

The delay in the emergence of resistance by mixing a high-risk fungicide with a low-risk fungicide occurs because the low-risk fungicide i) further decreases the size of the sensitive pathogen population and therefore the number of mutants generated per time unit, and ii) decreases the infection efficiency and therefore the survival probability of the resistant strain. The sensitivity of the emergence time to changes in the dose rate of the low-risk fungicide (for constant dose rates of the high-risk fungicide) is much less at higher dose rates, for similar reasons to those described for high-risk fungicides above.

### Comparison to the literature

Most experimental studies describe the development of fungicide resistance in response to different treatment strategies for the selection phase [4], [5], [6], [7], [8], [9], [10], [12]. To our knowledge, there are four experimental studies which report the evolution of fungicide resistance in a sensitive laboratory population. In these studies fungicide resistance either did not evolve [31], [32] or was already emerged (frequencies ≥1%) when detected [32], [33], [34]. The effect of fungicide treatment strategies on the time to the emergence of resistance can therefore not be determined from these studies.

There is modelling literature on the development of fungicide resistance in response to for example the dose rate, spray frequency and spray coverage of a fungicide [35], [36], [37], [38], [39], [40] and in response to concurrent, sequential, alternating or mixture use of fungicides [18], [19], [35], [40], [41], [42], [43]. The models in virtually all of these studies were deterministic and are therefore unable to account for the stochastic nature of the dynamics of the resistant strain in the emergence phase. To our knowledge, there are two modelling studies which describe the dynamics of the resistant strain during a part of the emergence phase as well as during the selection phase. In one of these studies [2], as is the case for our model, the dynamics of the resistant strain in the emergence phase were described using a stochastic model and the dynamics of the resistant strain during the selection phase were described using a deterministic model. However, contrary to our model, the emergence phase was assumed to last only until the first resistant mutant arose and the model did not therefore account for the possibility that a mutant may subsequently become extinct due to random processes. As a result, it was suggested that the length of the emergence phase increased with an increasing degree of control of the sensitive pathogen population by fungicides, which decreased the mutation rate. In our model, the mutation rate also decreases with increasing dose rates of fungicides (due to a smaller sensitive pathogen population), but the emergence time of resistance was predicted to decrease with increasing fungicide dose rates, due to the increased survival probability of resistant mutants at higher dose rates.

In the second modelling study [44], the resistant strain was introduced at the start of simulations and its dynamics in response to applications of a high-risk fungicide were described using a stochastic model. No clear distinction was made between the emergence phase and the selection phase and the effect of the dose rate of a fungicide on the length of the emergence time was not determined. The output of the model suggested that the resistant strain can only invade a sensitive pathogen population when the fitness of the resistant strain is high enough compared to the fitness of the sensitive strain in the presence of fungicides. Our model results show that resistance always emerged in a population of *M. graminicola* on winter wheat for all simulated dose rates of the high-risk fungicide, and scenarios for fitness costs of resistance and partial resistance. However, our model results show that decreasing the fitness of the resistant strain relative to the sensitive strain, by increasing fitness costs of resistance or increasing the sensitivity of the resistant strain to the high-risk fungicide, increases the emergence time. Further decreasing the relative fitness of the resistant strain in our model simulations decreases the probability of emergence below 100% (results not shown).

### Implications for resistance management

Hobbelen et al. [18] defined the effective life of a fungicide as the time from the start of a treatment until the loss of effective disease control. They subsequently used the term “effective life” to indicate the number of years that different treatment strategies were able to provide disease control in the selection phase only [18], [19]. However, the time from the start of a treatment until the loss of effective disease control above includes both the emergence phase and the selection phase. Below, we use the term “effective life” to indicate the emergence time plus the number of years that a fungicide can provide effective disease control in the selection phase.

When resistance is detected in the field for the first time, it is likely that the resistant strain is already present at a frequency of one percent or more in certain areas. To reach that point, the pathogen population has been evolving by emergence and selection for many generations. Hence, much of the opportunity to slow evolution has already been lost if anti-resistance strategies are put in place in response to detection. Strategies need to be implemented at introduction. This raises two new questions. Does the treatment strategy which is most effective at delaying resistance emergence differ from the treatment strategy which is most effective at slowing selection? If so, which of these two strategies should be used?

For solo use of a high resistance risk fungicide, the median emergence time of resistance was highest for the lowest dose rate of the high-risk fungicide that could provide effective control of an average epidemic of *M. graminicola* on winter wheat. Hobbelen et al. [18], [19] determined the effect of the dose rate of a high-risk fungicide on the number of years that a high-risk fungicide can provide effective disease control in the selection phase for the same host-pathogen system. Their analyses showed that this number of years was constant or slightly decreased with increasing dose rate of the high-risk fungicide. This result was consistent for a range of fitness costs of resistance and for different degrees of partial resistance. For solo use of a high-risk fungicide, the model output thus suggests that both the part of the effective life spent in the emergence phase and the part of the effective life spent in the selection phase can be maximised, for a fixed number of fungicide applications per crop, by using the lowest dose which can provide effective disease control.

For mixtures of a high-risk and a low-risk fungicide, the median emergence time of resistance to the high-risk fungicide was highest when high dose rates of the low-risk fungicide were combined with the lowest possible dose rate of the high-risk fungicide necessary to provide sufficient disease control of an average epidemic of *M. graminicola* on winter wheat. Hobbelen et al. [18] determined the number of years that mixtures of a low-risk and a high-risk fungicide can provide sufficient disease control in the selection phase for the same host-pathogen system. Similar to the emergence phase, their analysis shows that this number of years is highest when high dose rates of the low-risk fungicide are combined with the lowest possible dose rate of the high-risk fungicide necessary to provide sufficient disease control. It can be concluded that the dose and mixture treatment strategies which are most effective at delaying the evolution of fungicide resistance, do not differ between the emergence phase and the selection phase.

### Generality of the model assumptions

The specific model in this paper describes the emergence of resistance to a high-risk fungicide in *M. graminicola* populations on winter wheat. However, the structure and assumptions underlying the model apply to many foliar fungal pathogens of cereal crops. For example, only parameter values would need to be changed to describe the development of the canopy of cereal crops other than winter wheat. Similarly, the division of the life cycle of fungal pathogens into latent and infectious stages is representative of all fungal pathogens.

The division of our model into deterministic and stochastic sub-models is to some extent artificial, because stochastic processes will not only influence the dynamics of the resistant strain, but also the dynamics of the host and the sensitive pathogen population. However, such a division is justified when the density of the host and the sensitive pathogen population are so high during most of the growing season that extinction due to stochastic processes is highly unlikely. The advantage of using a deterministic instead of a stochastic model to describe large populations is the much shorter simulation time.

There are also a number of limitations to the generality of the model. Firstly, the sensitivity of pathogen strains is assumed to be constant in time. As a result, the model cannot be used to describe a quantitative type of resistance development, characterised by a gradual decrease in sensitivity of the pathogen population due to the accumulation of mutations over time. The best strategy for delaying the emergence of strains with sharply decreased sensitivity due to a single mutation may not be the best strategy for delaying the emergence of strains in which the reduction in sensitivity due to each mutation is relatively small. A second limitation is that the model does not account for the spatial variation in the treatment programs for fungicides. In reality, spores which are resistant to the fungicide which is applied in one area may disperse to neighbouring areas treated with another fungicide. If the spore is sensitive to the fungicide applied in the neighbouring area, it is unlikely to survive. Accounting for spatial variation may therefore decrease the survival probability of resistant mutants and increase the emergence time of resistance. Thirdly, in the absence of peer-reviewed data, we have assumed that the mutation probability is not increased by the exposure to fungicides. Finally, when a low-risk and a high-risk fungicide are applied in a mixture, we have assumed that both fungicides act independently on the life-cycle parameters of the pathogen strain. The last two assumptions can however be changed by small adjustments to the model equations.

### Priorities for future research

This initial analysis of fungicide resistance emergence opens several lines of future enquiry. Although several experiments have shown that environmental stress (caused for example by nutrient limitation, UV light, oxidative stress, antibiotic exposure or low pH) can increase the mutation rate in bacteria [45], [46], [47], [48], a recent review [3] found no published studies on the effect of the dose rate of fungicides on the probability of mutations which decrease the sensitivity of pathogens to fungicides. Future work should test if there is a relationship between dose rate and the probability of such mutations, as this may change the current conclusion that mixing a low-risk with a high-risk fungicide increases the emergence time.

It would be useful to develop a stochastic model that describes both the emergence phase and the selection phase in the evolution of fungicide resistance. This would allow the calculation of a distribution for the time from the introduction of a fungicide on the market to the loss of effective disease control due to the evolution of resistance. In addition, a spatial version of such a model would provide insight into spatial differences in the evolution of resistance. At the end of the emergence phase the number of resistant lesions in the pathogen population is very small and large areas will still be occupied by a completely sensitive pathogen population. The time from the introduction of a fungicide on the market to the loss of effective control will therefore differ between wheat growing regions, depending on the rate of dispersal.

So far, we have used our model to analyse the effect of the dose rate of a high-risk fungicide on the emergence time of resistance and the usefulness of mixing a low-risk and a high-risk fungicide for delaying the emergence of resistance to the high-risk fungicide. The usefulness of other anti-resistance strategies remains to be evaluated. Finally, more research is needed to determine the effect of exposure to a mixture of fungicides on the life-cycle parameters of pathogen strains. In this model, we have assumed that fungicides act independently on life-cycle parameters. Deviations from this assumption will change the efficacy of fungicide mixtures and therefore the size of the sensitive pathogen population, which in turn influences the mutation rate and the ability of mutant spores to survive.

### Model testing

In order to experimentally determine the emergence time of resistance, an emergence threshold must be defined above which the resistant strain is unlikely to become extinct if the fungicide treatment continues. The emergence threshold in our model was defined as the number of resistant lesions at the start of a growing season giving a specified low probability of extinction in the absence of new mutations. In experiments, it would not be possible to use this threshold as the generation of new mutations by the sensitive pathogen population cannot be stopped. It is therefore difficult to experimentally determine the length of the emergence phase in the evolution of resistance. However, it may be possible to design experiments that determine the time that it takes for a resistant strain to arise in a completely sensitive pathogen population and subsequently invade this population until it constitutes a specified very small threshold frequency in the pathogen population. It is important to note that the emergence threshold is a number of resistant lesions, which applies irrespective of the size of the sensitive pathogen population. For small pathogen populations, the emergence threshold corresponds to a higher frequency of the resistant strain in the pathogen population than for a large pathogen population and the required sample size to detect the resistant strain early may be less. However, for smaller populations, the time until a resistant mutant arises will be longer than for a large pathogen population.

### Conclusion

In this study, we formulated a model structure to describe the emergence of resistance in a sensitive pathogen population. The resistance simulated was representative of observed cases where a mutation affecting the target protein results in a large shift in sensitivity. We subsequently showed how the model could be used to evaluate the usefulness of treatment strategies for delaying the emergence of such resistance. There are important conclusions from the model output which have implications for practical resistance management. In the absence of previous exposure to high-risk fungicides with the same mode of action, the model output suggests that resistance to high-risk fungicides is likely to emerge after their introduction on the market, making it important that anti-resistance strategies implemented at introduction are effective against both emergence and selection. Our analysis suggests that the dose and mixture treatment strategies which have been shown previously to reduce selection for resistance in the selection phase, may also be effective in prolonging the emergence phase in the evolution of resistance to fungicides.

## Supporting Information

Text S1 **Robustness of model outputs (emergence time) to structural model changes (fungicide decay rate, definition of emergence threshold).**
(DOCX)
