# Supplementary material for: The Emergence of Resistance to Fungicides
Source: PLoS One. 2014 Mar 21;9(3):e91910. doi: 10.1371/journal.pone.0091910 (PMC3962370; doi:10.1371/journal.pone.0091910)
Supplement: Text S1 — Robustness of model outputs (emergence time) to structural model changes (fungicide decay rate, definition of emergence threshold). (DOCX) [file pone.0091910.s001.docx]

**Supporting Information.**

**Text S1: Robustness of model outputs (emergence time) to structural model changes (fungicide decay rate, definition of emergence threshold).**

The second part of the supporting information explores the effect of using an alternative function (Equations S1) to describe the decay of the activity of fungicides on the emergence time (Figure S1). The results show that the emergence time shows the same qualitative patterns for the two investigated functions (Figures S2 to S6), although there were quantitative differences.

The second part of the supporting information considers the effect of the definition of emergence threshold on emergence time (Figures S7 through to S11). The results show that the emergence time shows the same qualitative and quantitative pattern for the two definitions investigated.

The last figure in the supplementary materials (Figure S12) shows the effect of the number of model simulations on the estimated median and mean emergence time. The figure shows that the number of simulation runs used to construct the figures in the main text is sufficient to estimate means and medians accurately.

**Equations S1. Alternative equations used to describe the decay of fungicides in time.**

In the default scenario, we described the time interval from application until the loss of activity of the active compounds in fungicides by an exponential distribution (Equations 10 and 11). To explore the effect of the type of distribution for the time interval from application until the loss of activity of the active compounds in fungicides, we also determined emergence times using a gamma distribution for this time interval. The gamma distribution is characterized by scale parameter $\xi$ and shape parameter $k$. We calculated parameter $\xi$ such that the mean of the gamma distribution (k$\xi$) was the same as the mean of the exponential distribution used in the default scenario ($1/\nu$). We set $k$=5 to obtain a gamma distribution which resembles a positive Gaussian distribution with a peak close to the mean active period of fungicides. In contrast to the exponential distribution, using this gamma distribution assumes that the loss rate of fungicide activity is initially low after application, then peaks and subsequently decreases again.

We used the “linear chain trick” [Lloyd AL (2001) Destabilization of epidemic models with the inclusion of realistic distributions of infectious periods. Proc. Biol. Sci. 268: 985–993] to change the exponential distribution of the time from application until the loss of activity of the active compounds fungicides to a gamma distribution. This leads to the following equations for the concentrations of the low-risk ($C_{A}$) fungicide:

$$\frac{dC_{A,1}}{dt}=-k\nu_{A}C_{A,1}$$

$$\frac{dC_{A,i}}{dt}={k\nu}_{A}C_{A,(i-1)}-k\nu_{A}C_{A,i}$$

$C_{A}=\sum_{i=1}^{i=k} C_{A,i}$.

Similarly, the equations for concentration of the high-risk ($C_{B}$) are:

$$\frac{dC_{B,1}}{dt}=-k\nu_{B}C_{B,1}$$

$$\frac{dC_{B,i}}{dt}={k\nu}_{B}C_{B,(i-1)}-k\nu_{B}C_{B,i}$$

$C_{B}=\sum_{i=1}^{i=k} C_{B,i}$.

In these equations, parameter $k$ represents the shape parameter of the gamma distribution.

A change in the function used to describe the decay of fungicide activity affects the disease control by fungicides at a given dose rate. We therefore obtained new estimates for the values of dose-response curve parameters (Equations 8, 9, 19 and 20) by fitting the model which assumes a gamma distribution for the time interval from application until the loss of activity of the active compounds in fungicides, to the same data that was used to determine the values of these parameters for the default model [Hobbelen PHF, Paveley ND, van den Bosch F (2011) Delaying selection for fungicide insensitivity by mixing fungicides at a low and high risk of resistance development: a modelling analysis. Phytopathology 101: 1224-1233.]. This resulted in the following parameter estimates: $\alpha_{A,s}$=0.73, $\beta_{A,s}$=5.6, $\alpha_{B,s}$=1 and $\beta_{B,s}$=$\beta_{B,r}$=44.8 ($\alpha_{B,r}$=variable, see text).

**
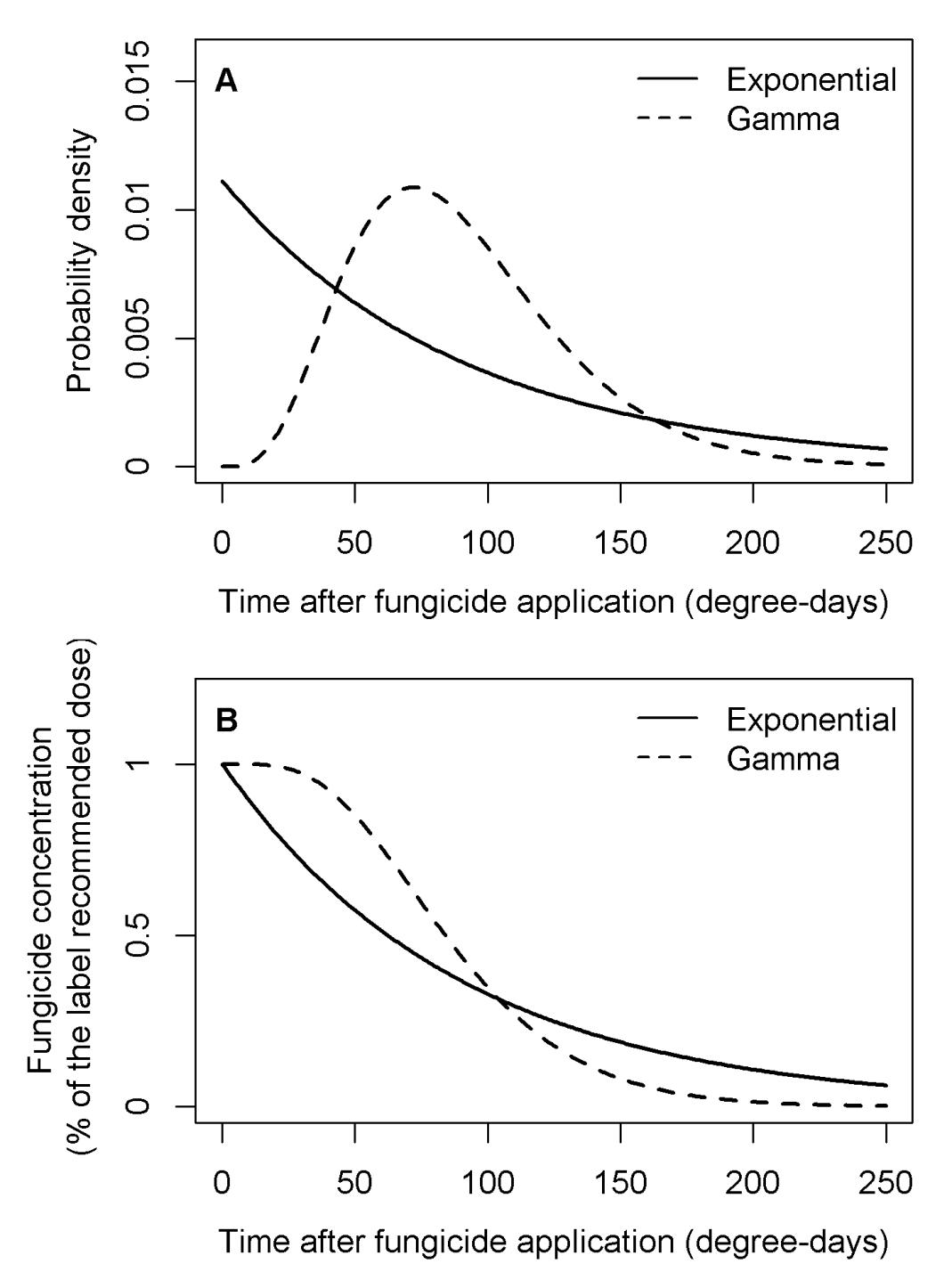
**

**Figure S1. Fungicide decay curves.**

A comparison of the two functions used in the model to describe the decay of fungicides in time. Panel A shows the probability density functions of the exponential (default) and gamma distributions for the time interval from application until the loss of activity of fungicides. Panel B shows the corresponding decay of the fungicide concentrations in time.


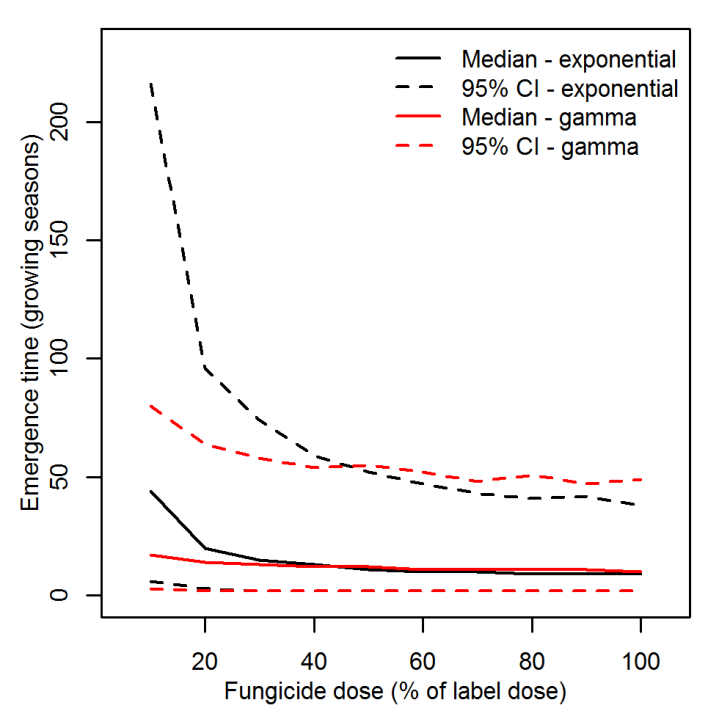


**Figure S2. Emergence time for different fungicide decay curves, dose effects.**

The effect of the shape of the curve describing the decay of fungicides in time on the emergence time of resistance for the default scenario. The emergence time of resistance in a sensitive population of *M. graminicola* on winter wheat was determined for different dose rates of the high-risk. We compared emergence times calculated assuming an exponential distribution of the time interval from application until the loss of activity of fungicides with emergence times calculated assuming a gamma distribution for this time interval. The default scenario assumes that i) fitness costs of resistance reduce the infection efficiency by 10%, ii) resistance to the high-risk is complete and iii) the mutation probability amounts to 1.13∙10^-16^.

**
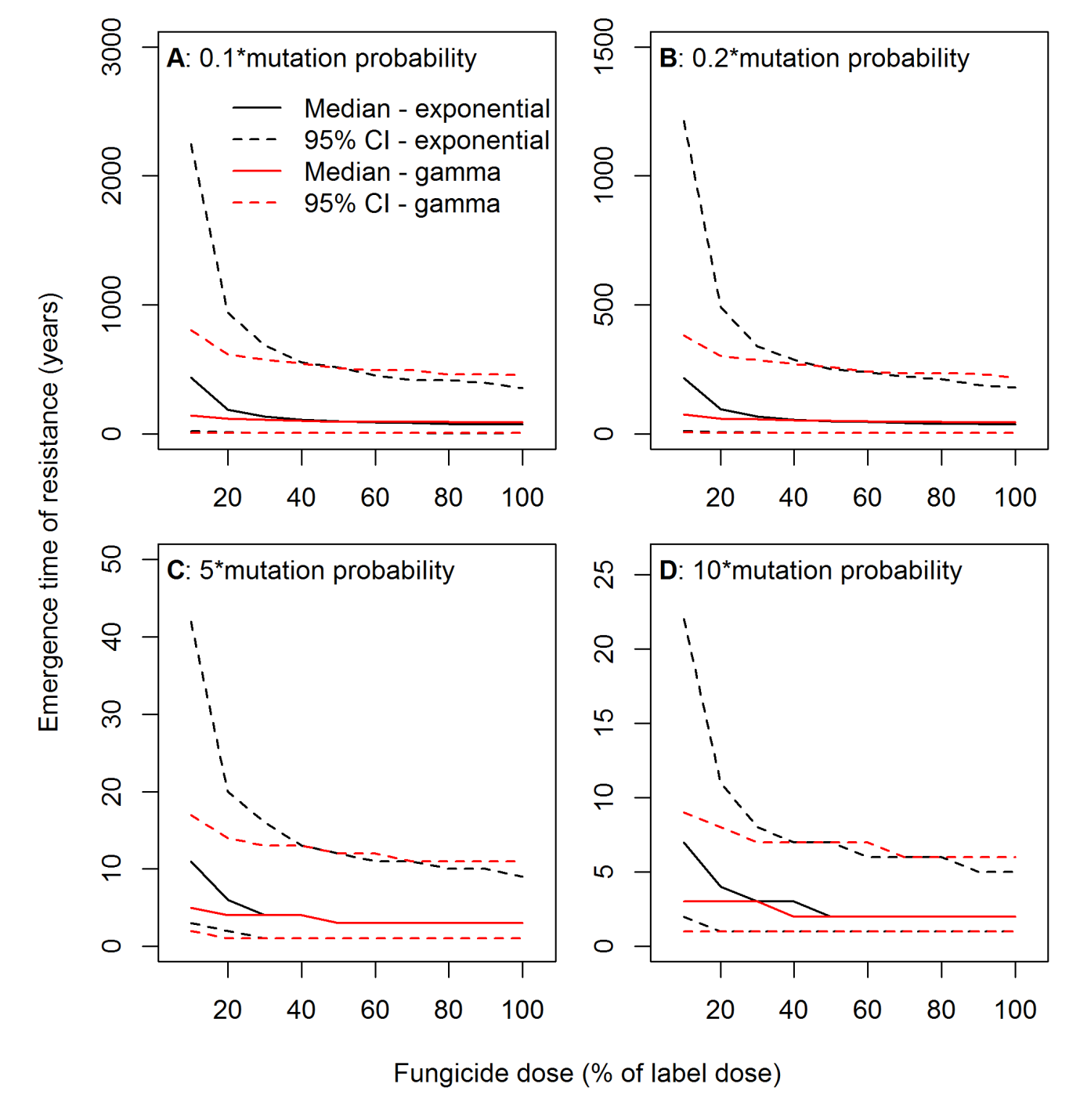
**

**Figure S3. Emergence time for different fungicide decay curves, mutation rate effects.**

The effect of the shape of the curve describing the decay of fungicides in time on the emergence time of resistance for different values of the mutation probability. The emergence time of resistance in a sensitive population of *M. graminicola* on winter wheat was determined for different dose rates of the high-risk fungicide for mutation probabilities amounting to 0.1, 0.2, 5 and 10 (A-D) times the default value of 1.13∙10^-16^. We compared emergence times calculated assuming an exponential distribution for the time interval from application until the loss of activity of fungicides with emergence times calculated assuming a gamma distribution for this time interval. Fitness costs of resistance were assumed to reduce the infection efficiency by 10% and resistance to the high-risk was assumed to be complete.

**
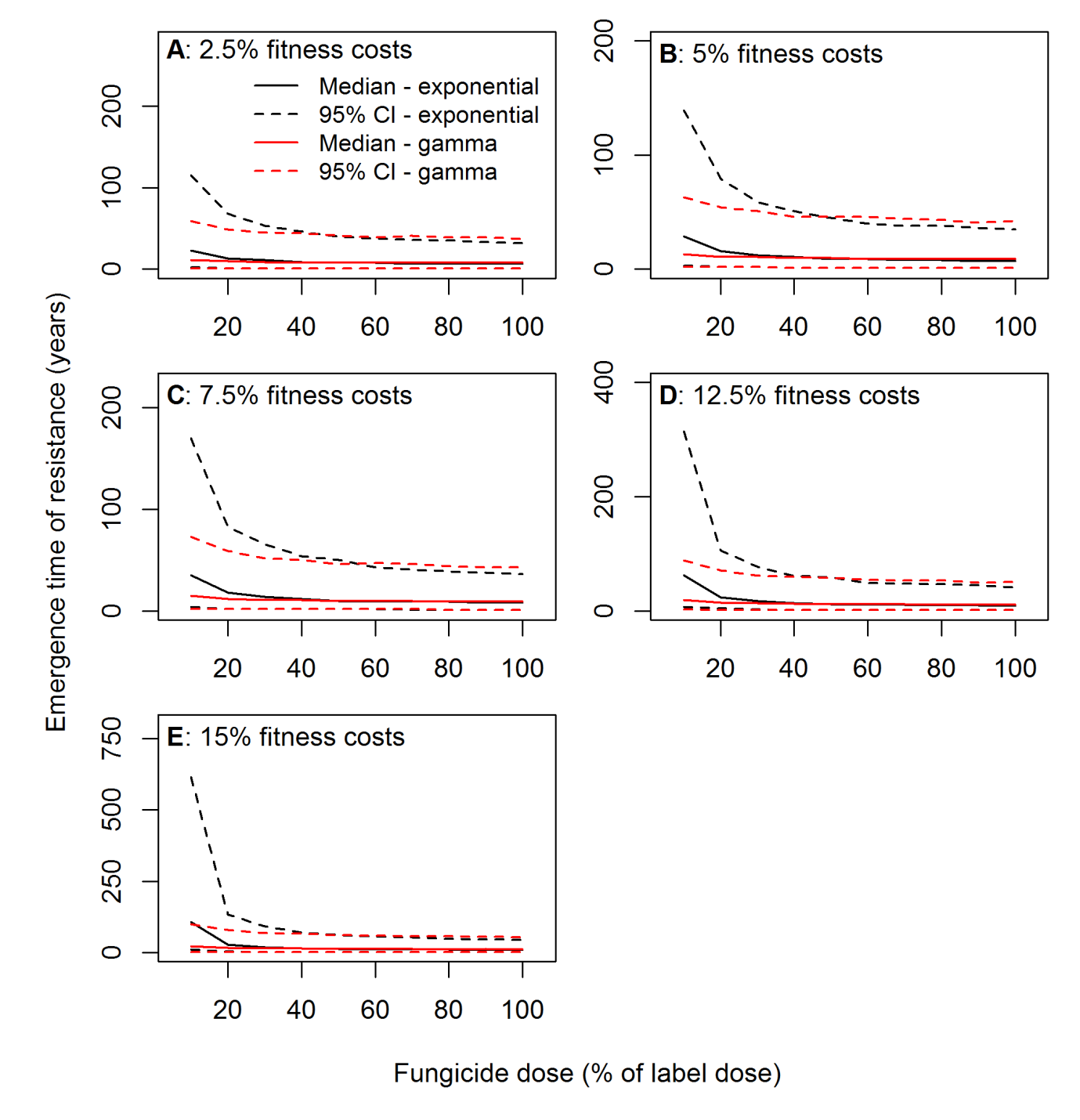
**

**Figure S4. Emergence time for different fungicide decay curves, fitness cost effects.**

The effect of the shape of the curve describing the decay of fungicides in time on the emergence time of resistance for different values of the fitness costs of resistance. The emergence time of resistance in a sensitive population of *M. graminicola* on winter wheat was determined for different dose rates of the high-risk fungicide for fitness costs of resistance amounting to 2.5%, 5%, 7.5%, 12.5% and 15% (A-E). We compared emergence times calculated assuming an exponential distribution of the time interval from application until the loss of activity of fungicides with emergence times calculated assuming a gamma distribution for this time interval. The mutation probability was set to 1.13∙10^-16^ and resistance to the high-risk was assumed to be complete.

**
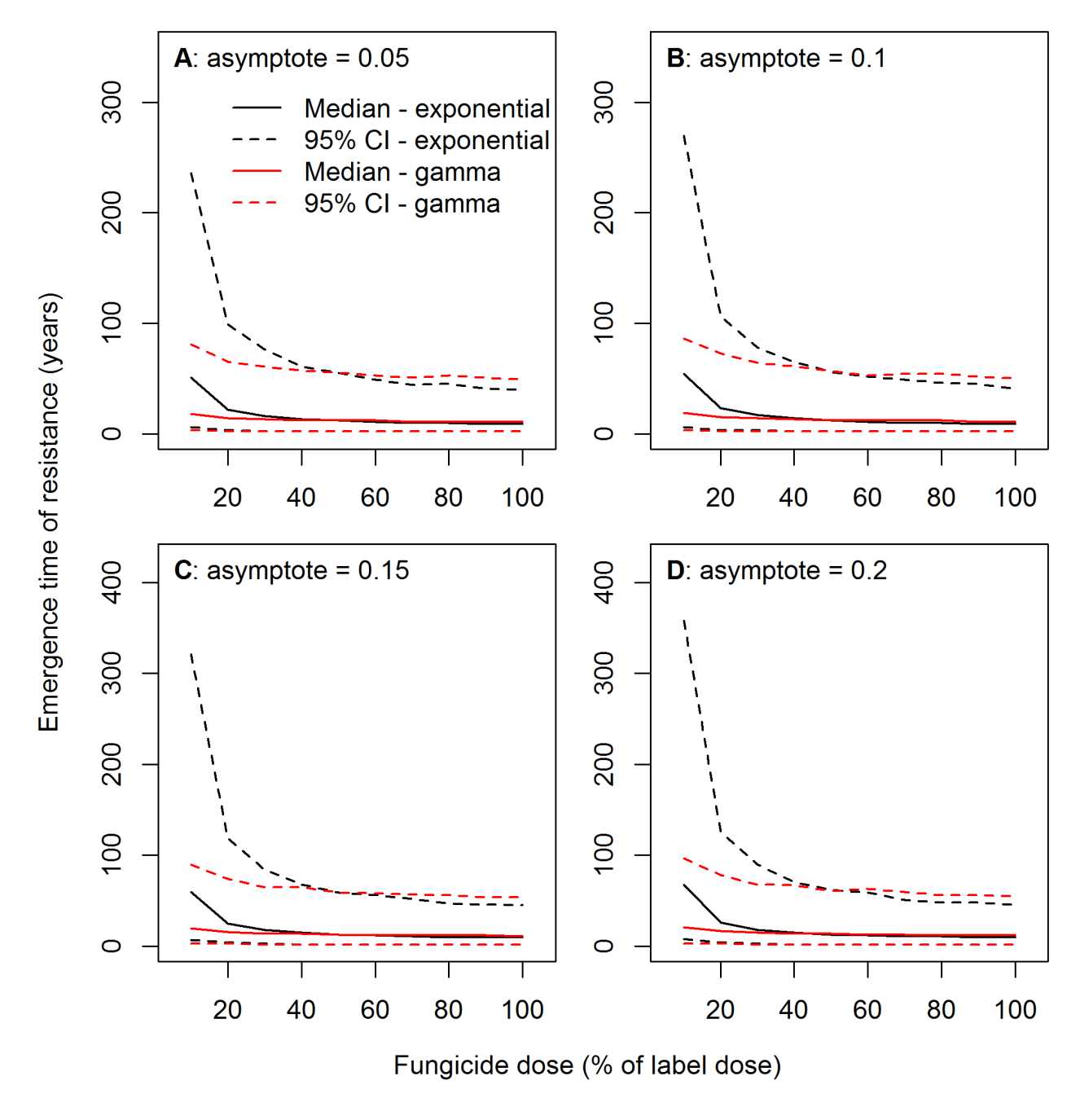
**

**Figure S5. Emergence time for different fungicide decay curves, partial resistance.**

The effect of the shape of the curve describing the decay of fungicides in time on the emergence time of resistance for different levels of partial resistance. The emergence time of resistance in a sensitive population of *M. graminicola* on winter wheat was determined for different dose rates of the high-risk fungicide for different levels of partial resistance. We compared emergence times calculated assuming an exponential distribution of the time interval from application until the loss of activity of fungicides with emergence times calculated assuming a gamma distribution for this time interval. The level of partial resistance was varied by adjusting the asymptote of the dose-response curve which determines the sensitivity of the resistant strain to the high-risk fungicide. We calculated emergence times for values of this asymptote amounting to 0.05, 0.1, 0.15 and 0.2 (A-D). We compared emergence times calculated using a constant emergence threshold of 30 resistant lesions at the start of a growing season with emergence times calculated using a variable emergence threshold. The variable emergence threshold was defined as the lowest possible number of resistant lesions at the start of a growing season for which the probability of the resistant strain becoming extinct during a period of 100 years in the absence of new mutations was < 5%.


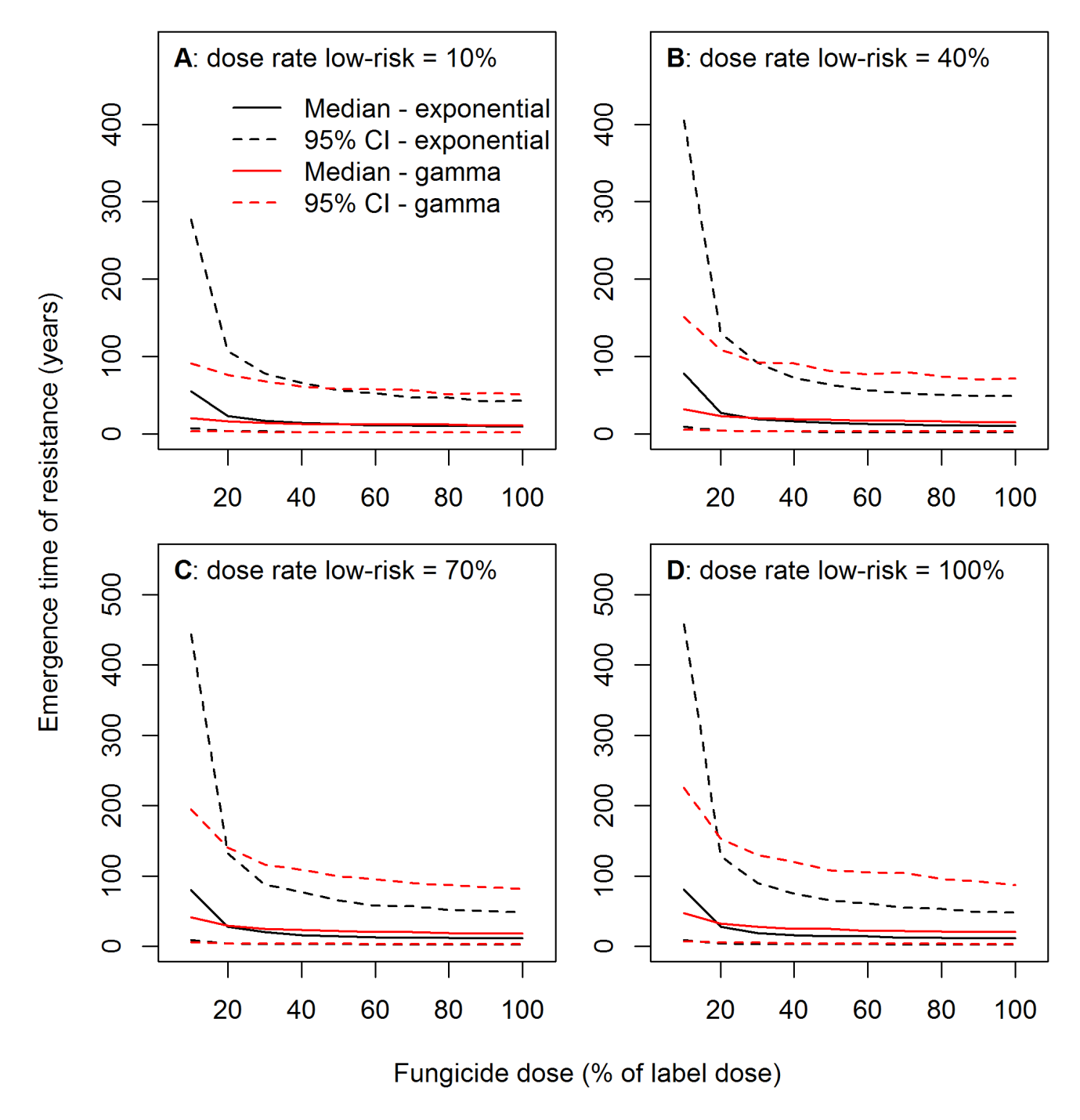


**Figure S6. Emergence time for different fungicide decay curves, dose rate effects.**

The effect of the shape of the curve describing the decay of fungicides in time on the emergence time of resistance to a high-risk fungicide when mixed with a low-risk fungicide. The emergence time of resistance in a sensitive population of *M. graminicola* on winter wheat was determined as a function of the dose rate of the high-risk fungicide when mixed with a low-risk fungicide applied at dose rates amounting to 10%, 40%, 70% and 100% (A-D) of the label recommended dose. We compared emergence times calculated assuming an exponential distribution of the time interval from application until the loss of activity of fungicides with emergence times calculated assuming a gamma distribution for this time interval. Simulations were performed for the default which assumes that i) fitness costs of resistance reduce the infection efficiency by 10%, ii) resistance to the high-risk is complete and iii) the mutation probability amounts to 1.13∙10^-16^.


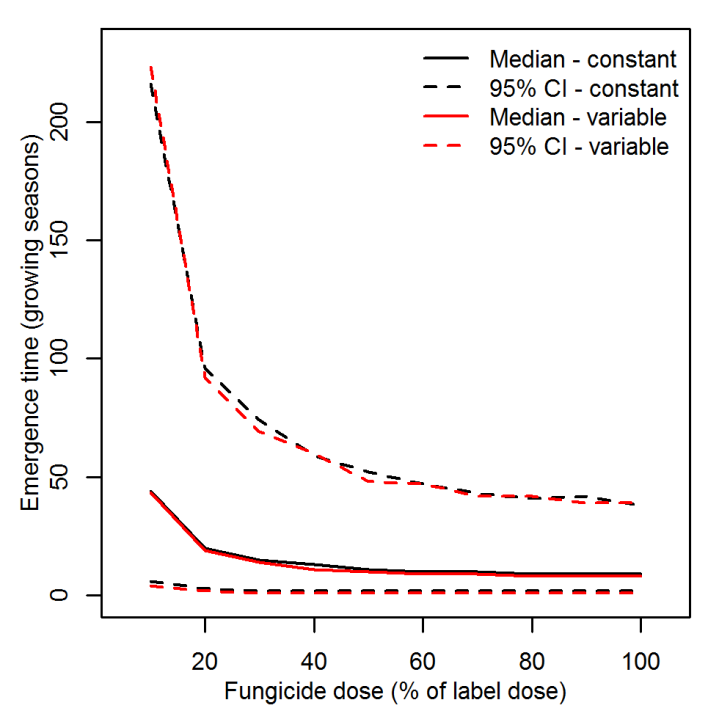


**Figure S7. Emergence time and the definition of the emergence threshold, dose effects.**

The effect of the definition of the threshold for emergence on the emergence time of resistance for the default scenario. The emergence time of resistance in a sensitive population of *M. graminicola* on winter wheat was determined for different dose rates of the high-risk fungicide using two definitions of the emergence threshold. We compared emergence times calculated using a constant emergence threshold of 30 resistant lesions at the start of a growing season with emergence times calculated using a variable emergence threshold. The variable emergence threshold was defined as the lowest possible number of resistant lesions at the start of a growing season for which the probability of the resistant strain becoming extinct during a period of 100 years in the absence of new mutations was < 5%. The default scenario assumes that i) fitness costs of resistance reduce the infection efficiency by 10%, ii) resistance to the high-risk is complete and iii) the mutation probability amounts to 1.13∙10^-16^.


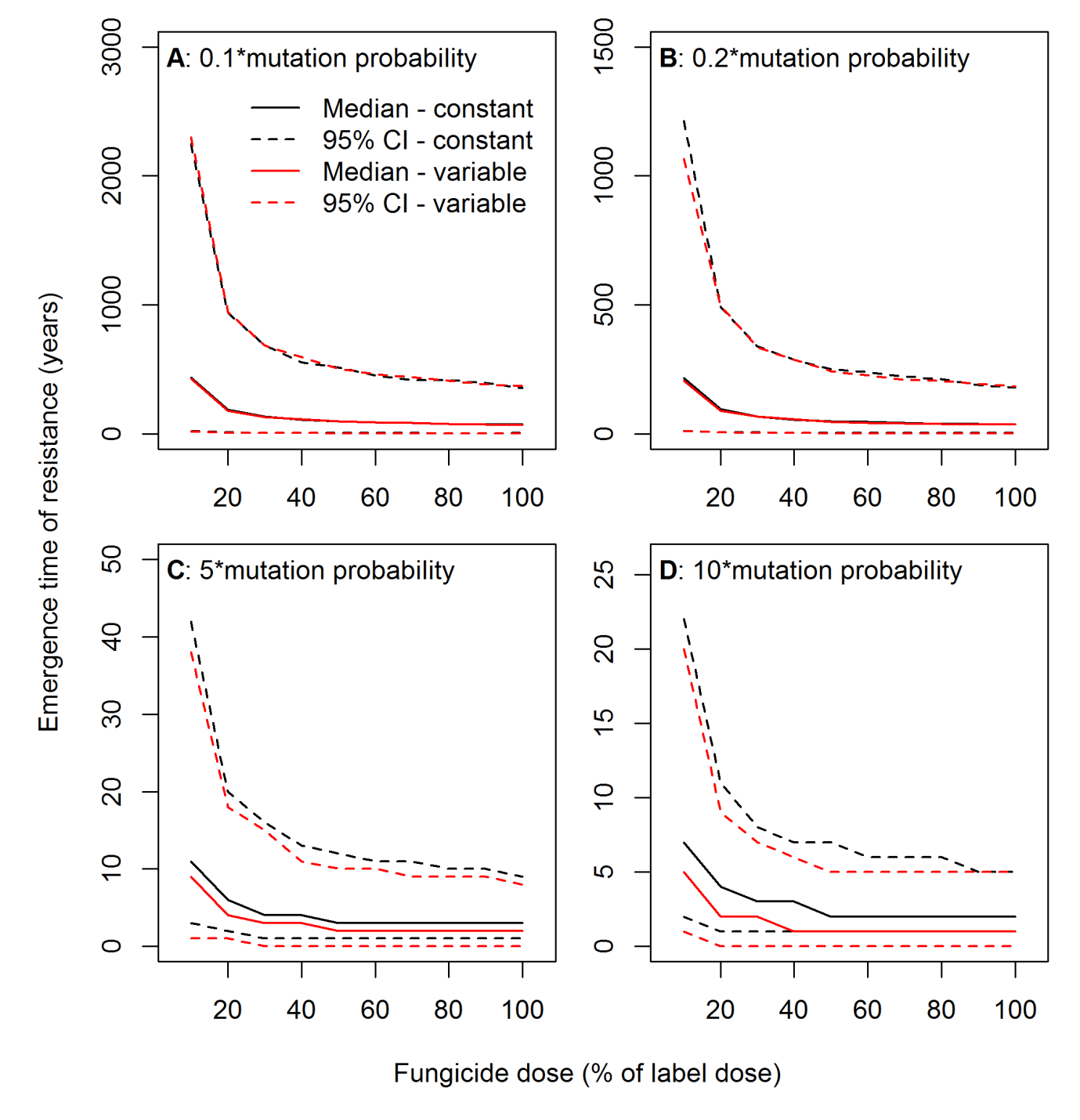


**Figure S8. Emergence time and definition of emergence threshold, mutation rate effects.**

The effect of the definition of the threshold for emergence on the emergence time of resistance for different values of the mutation probability. The emergence time of resistance in a sensitive population of *M. graminicola* on winter wheat was determined for different dose rates of the high-risk fungicide using two definitions of the emergence threshold for mutation probabilities amounting to 0.1, 0.2, 5 and 10 (A-D) times the default value of 1.13∙10^-16^. We compared emergence times calculated using a constant emergence threshold of 30 resistant lesions at the start of a growing season with emergence times calculated using a variable emergence threshold. The variable emergence threshold was defined as the lowest possible number of resistant lesions at the start of a growing season for which the probability of the resistant strain becoming extinct during a period of 100 years in the absence of new mutations was < 5%. Fitness costs of resistance were assumed to reduce the infection efficiency by 10% and resistance to the high-risk was assumed to be complete.

**
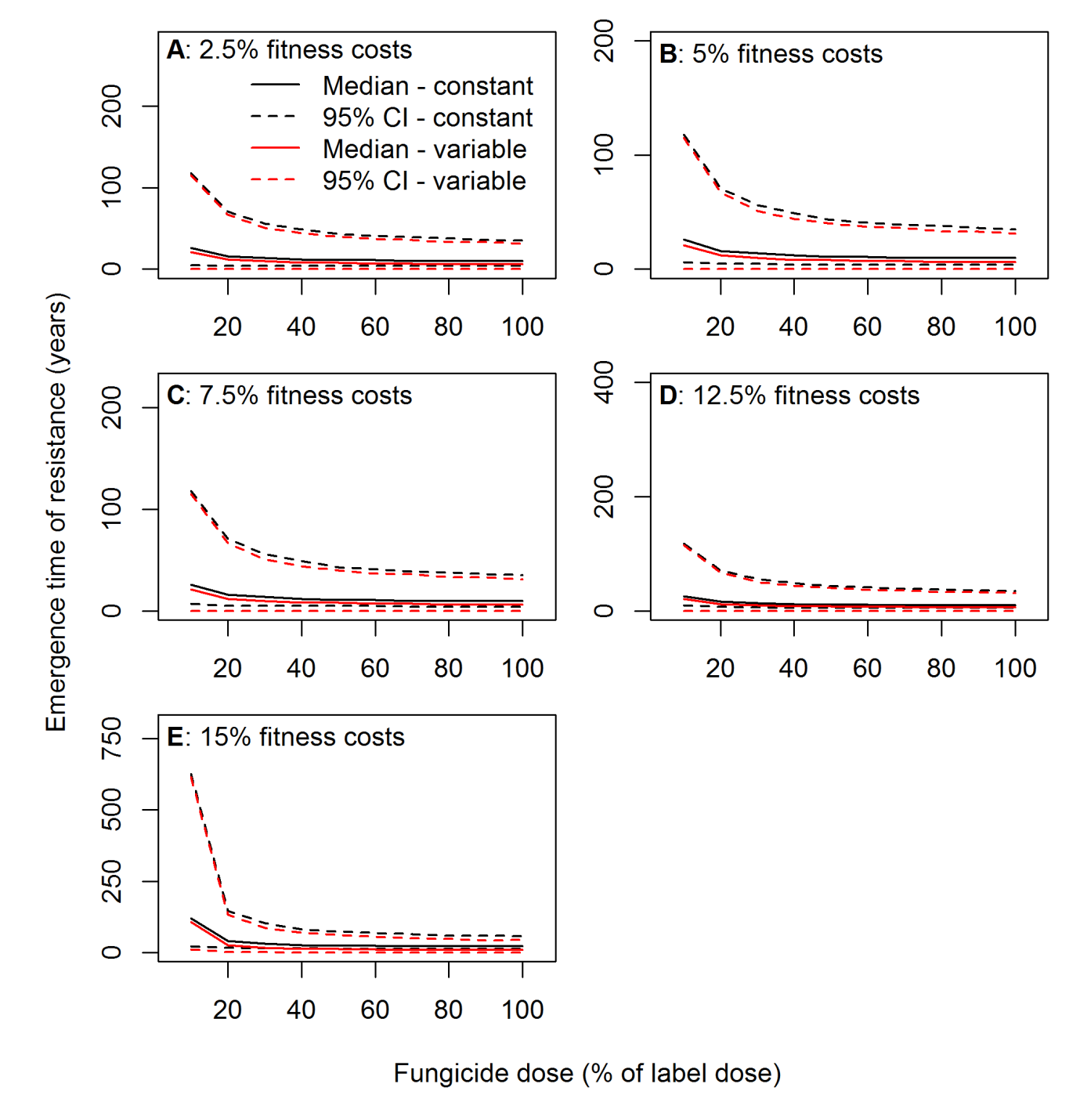
**

**Figure S9. Emergence time and definition of emergence threshold, fitness cost effects.**

The effect of the definition of the threshold for emergence on the emergence time of resistance for different values of the fitness costs of resistance. The emergence time of resistance in a sensitive population of *M. graminicola* on winter wheat was determined for different dose rates of the high-risk fungicide using two definitions of the emergence threshold for fitness costs of resistance amounting to 2.5%, 5%, 7.5%, 12.5% and 15% (A-E). We compared emergence times calculated using a constant emergence threshold of 30 resistant lesions at the start of a growing season with emergence times calculated using a variable emergence threshold. The variable emergence threshold was defined as the lowest possible number of resistant lesions at the start of a growing season for which the probability of the resistant strain becoming extinct during a period of 100 years in the absence of new mutations was < 5%. The mutation probability was set to 1.13∙10^-16^ and resistance to the high-risk was assumed to be complete.

**
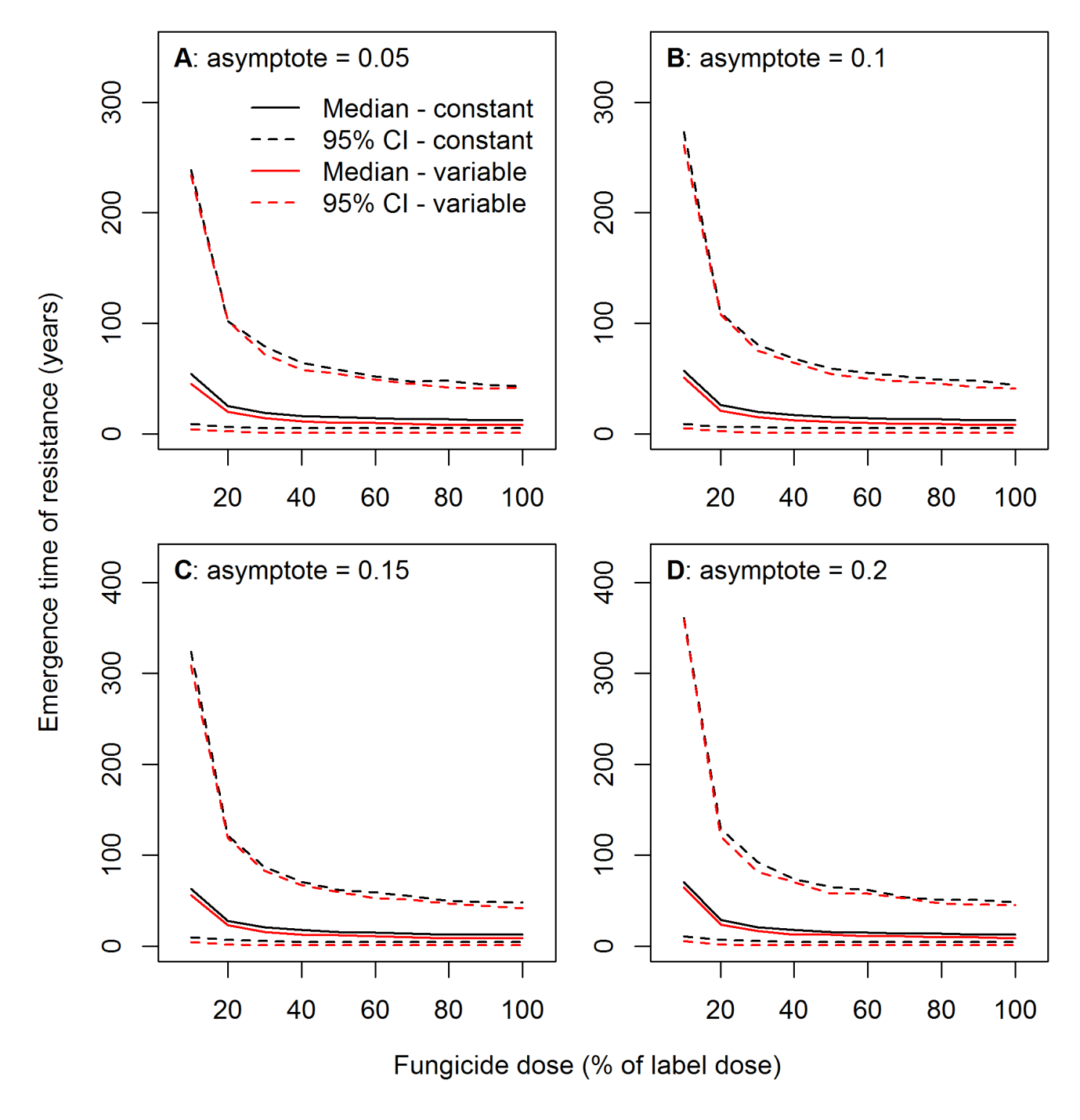
**

**Figure S10. Emergence time and definition of emergence threshold, dose response curve effects.**

The effect of the definition of the threshold for emergence on the emergence time of resistance for different levels of partial resistance. The emergence time of resistance in a sensitive population of *M. graminicola* on winter wheat was determined for different dose rates of the high-risk fungicide using two definitions of the emergence threshold for different levels of partial resistance. The level of partial resistance was varied by adjusting the asymptote of the dose-response curve which determines the sensitivity of the resistant strain to the high-risk fungicide. We calculated emergence times for values of this asymptote amounting to 0.05, 0.1, 0.15 and 0.2 (A-D). We compared emergence times calculated using a constant emergence threshold of 30 resistant lesions at the start of a growing season with emergence times calculated using a variable emergence threshold. The variable emergence threshold was defined as the lowest possible number of resistant lesions at the start of a growing season for which the probability of the resistant strain becoming extinct during a period of 100 years in the absence of new mutations was < 5%. The mutation probability was set to 1.13∙10^-16^ and resistance to the high-risk was assumed to be complete.

**
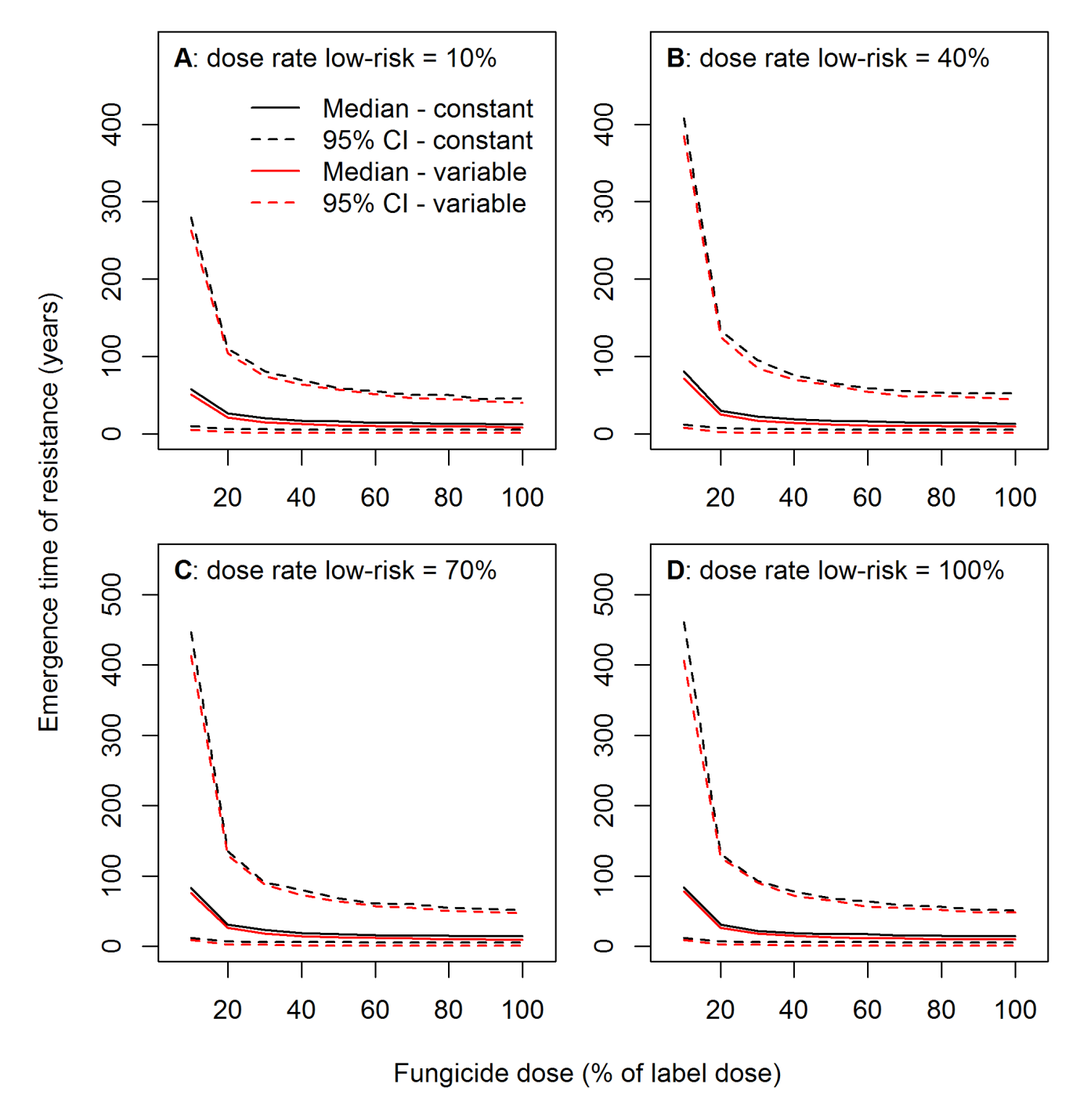
**

**Figure S11. Emergence time and definition of emergence threshold, partial resistance.** The effect of the definition of the threshold for emergence on the emergence time of resistance to a high-risk fungicide when mixed with a low-risk fungicide. The emergence time of resistance in a sensitive population of *M. graminicola* on winter wheat was determined as a function of the dose rate of the high-risk fungicide when mixed with a low-risk fungicide applied at dose rates amounting to 10%, 40%, 70% and 100% (A-D) of the label recommended dose. We compared emergence times calculated using a constant emergence threshold of 30 resistant lesions at the start of a growing season with emergence times calculated using a variable emergence threshold. The variable emergence threshold was defined as the lowest possible number of resistant lesions at the start of a growing season for which the probability of the resistant strain becoming extinct during a period of 100 years in the absence of new mutations was < 5%. Simulations were performed for the default which assumes that i) fitness costs of resistance reduce the infection efficiency by 10%, ii) resistance to the high-risk is complete and iii) the mutation probability amounts to 1.13∙10^-16^.

**
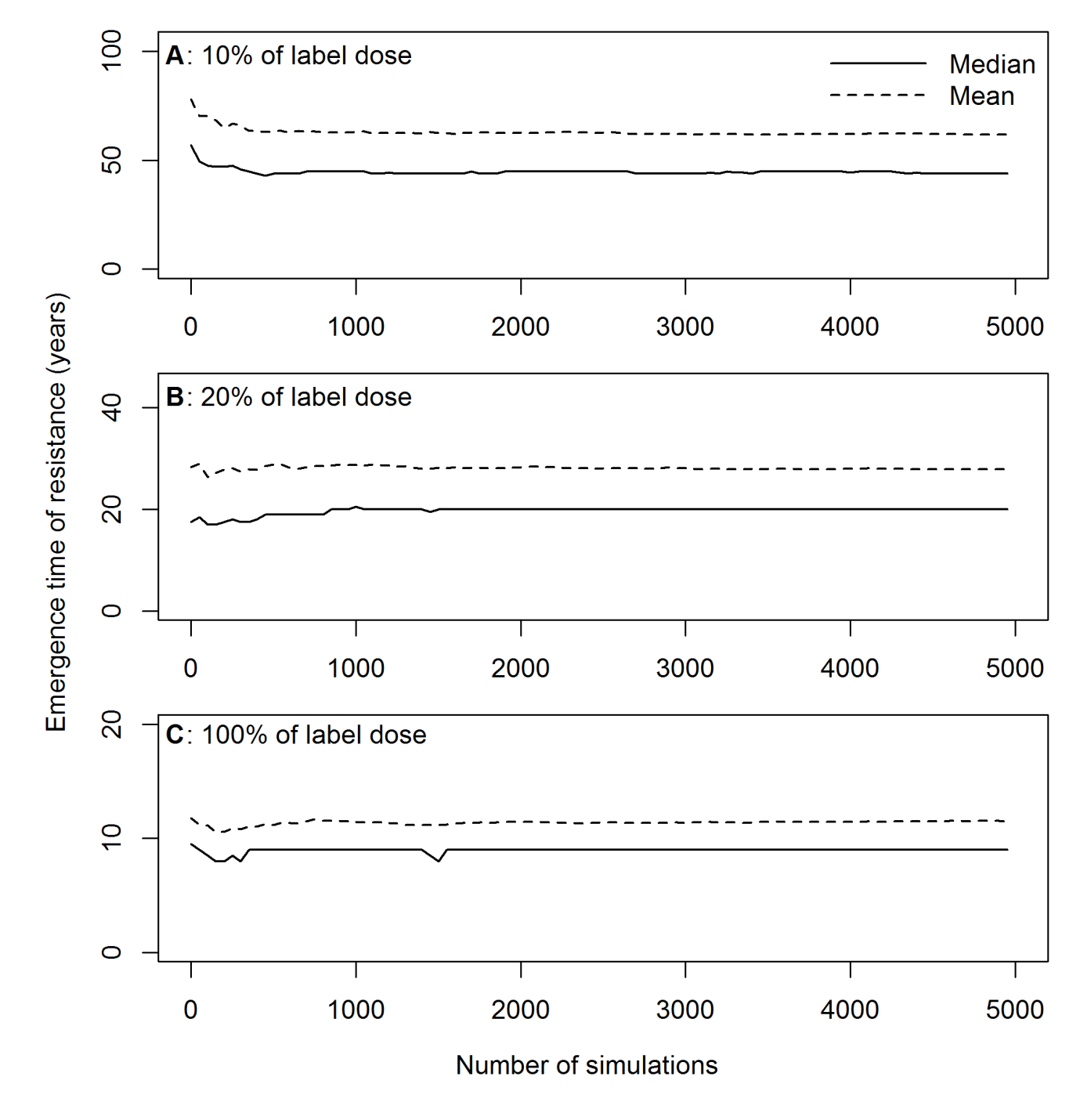
**

**Figure S12. Emergence time and number of simulation runs.**

The effect of the number of model simulations on the median and mean emergence time of resistance. The emergence time of resistance in a sensitive population of *M. graminicola* on winter wheat was determined for dose rates of the high-risk fungicide amounting to 10%, 20% and 100% (A-C) of the label dose. Simulations were performed using the default scenario which assumes that i) fitness costs of resistance reduce the infection efficiency by 10%, ii) resistance to the high-risk is complete and iii) the mutation probability amounts to 1.13∙10^-16^.
